# Supplementary material for: Simplified 0+1 and 1+1 pneumococcal vaccine schedules in Ho Chi Minh City, Vietnam: protocol for a randomised controlled trial
Source: BMJ Open. 2021 Nov 29;11(11):e056505. doi: 10.1136/bmjopen-2021-056505 (PMC8634020; doi:10.1136/bmjopen-2021-056505)
Supplement: Supplementary data [file bmjopen-2021-056505supp001.pdf]

APPENDICES

Table of contents

APPENDIX 1: Administrative information 2

APPENDIX 2: Sample collection and processing 5

1 SAMPLE COLLECTION 5

2 LABORATORY EVALUATIONS OF NASOPHARYNGEAL SWABS 6

2.1 TRADITIONAL CULTURE METHODS 6
2.2 QPCR AND MICROARRAY 7

3 LABORATORY EVALUATIONS OF BLOOD SAMPLES 7

3.1 ELISAS 7
3.2 OPAS 8
3.3 B CELL ASSAYS 8

4 REFERENCES 9

APPENDIX 3: Information statement and Consent form 10

PART I: INFORMATION STATEMENT 10

PART II: CONSENT FORM 14

APPENDIX 1
Administrative information

Title
A randomised controlled trial of simplified 0+1 and 1+1 pneumococcal vaccine schedules in Ho Chi Minh City, Vietnam

Trial registration
ClinicalTrials.gov: NCT03098628

Trial registration - data set

Data category	Information
Primary registry and trial identifying number	ClinicalTrials.gov NCT03098628
Date of registration in primary registry	1 March 2017
Secondary identifying numbers	HREC36027
Source(s) of monetary or material support	Bill & Melinda Gates Foundation
Primary sponsor	Murdoch Children’s Research Institute, Australia
Contact for public queries	Professor Kim Mulholland kim.mulholland@lshtm.ac.uk
Contact for scientific queries	Professor Kim Mulholland kim.mulholland@lshtm.ac.uk
Public title	A randomised controlled trial of simplified 0+1 and 1+1 pneumococcal vaccine schedules in Ho Chi Minh City, Vietnam
Scientific title	Trial of simplified pneumococcal vaccination in Vietnam II (VPT-II): the herd immunity approach
Countries of recruitment	Vietnam
Health condition(s) or problem(s) studied	Pneumococcal vaccination responses
Intervention(s)	Active Comparator V: PCV10 administered at 12 months of age (0+1 PCV10)
	Active Comparator W: PCV13 administered at 12 months of age (0+1 PCV13)
	Active Comparator X: PCV10 administered at 2 and 12 months of age (1+1 PCV10)
	Active Comparator Y: PCV13 administered at 2 and 12 months of age (1+1 PCV13)
	Control Z: PCV10 administered at end of trial (24 months)

Key inclusion and exclusion criteria	Inclusion criteria:<br>• aged between 2 months and 2 months plus 2 weeks<br>• no significant clinical maternal or perinatal history<br>• born at or after 36 weeks' gestation<br>• written and signed informed consent from parent/legal guardian<br>• lives within approximately 30 minutes of the commune health centre<br>• family anticipates living in the study area for the next 22 months
	Exclusion criteria:<br>• known allergy to any component of the vaccine<br>• allergic reaction or anaphylactic reaction to any previous vaccine<br>• known immunodeficiency disorder<br>• known HIV-infected mother<br>• known thrombocytopenia or coagulation disorder<br>• administration or planned administration of any immunoglobulin or blood product since birth<br>• severe birth defect requiring ongoing medical care<br>• chronic or progressive disease; seizure disorder<br>• history of severe illness<br>• receipt of any 2 month vaccines through the EPI program<br>• family plans on giving the infant *Quinvaxem* (DTP-Hib-HBV)
Study type	Interventional, randomised, parallel group, open label phase II/III trial. Outcome assessors (laboratory) blinded. Purpose: prevention.
Enrolment period	8 March 2017 – 11 June 2020
Sample size	Target: 2500<br>Number enrolled: 2501
Recruitment status	Active, not recruiting
Primary outcome	Vaccine-type (VT) pneumococcal carriage at 24 months of age
Key secondary outcomes	• VT pneumococcal carriage at 6, 12 and 18 months of age<br>• Non-VT pneumococcal carriage at 6, 12, 18 and 24 months of age<br>• Carriage of any pneumococcal serotype at 6, 12, 18 and 24 months of age<br>• Serotype-specific IgG antibody concentrations post-2-month dose, pre-12-month dose, post-12-month dose of PCV and at 24 months of age<br>• Serotype-specific opsonophagocytic indices pre- and post-12-month dose of PCV<br>• Serotype-specific memory B cell numbers pre- and post-12-month dose of PCV and at 24 months of age
Ethics Review	Approved by the Human Research Ethics Committee of the Royal Children's Hospital Melbourne and the Vietnam Ministry of Health Ethics Committee

Protocol version

Protocol version 5.0 dated 8 February 2018

Revision chronology
Original: Version 3.2, 11 October 2016.
First amendment: Version 4.0, 16 May 2017. Main reason for amendment: minor clarifications requested by the Vietnam Ministry of Health, along with a change to a final version number
Second amendment: Version 5.0, 8 February 2018. Main reason for amendment: to remove references to Japanese Encephalitis Vaccine (JEV) and measles-rubella (MR) vaccine, as for logistical reasons these are to be administered through the Commune Health Centres and not as part of the study

Roles and responsibilities

Sponsor contact information
Trial Sponsor: Murdoch Children's Research Institute, Royal Children's Hospital, Flemington Road, Parkville, Victoria 3052, Australia
Telephone: +61 3 8341 6200
Contact name: Professor Kim Mulholland

Other Institutions
Menzies School of Health Research, Darwin, Australia
Role: oversight of traditional microbiology (culture methods)

Family Health International (FHI360), Ho Chi Minh City, Vietnam
Role: external clinical trial monitoring

Bill & Melinda Gates Foundation
Role: funding source
The funder does not have any role in the trial conduct, trial management, laboratory tests, or data analyses.

APPENDIX 2
Sample collection and processing

1 Sample Collection

Participants will provide four (Groups V-Y) or two (Group Z) nasopharyngeal (NP) swabs over the course of the trial. Additionally, participants in the immunology sub-study will provide three (Groups V-Y) or one (Group Z) venous blood sample(s) over the course of the trial. A minimum of two members of the study team will be present during the collection of NP swabs and blood samples, to ensure no injury is caused by an infant's sudden movement. Distraction techniques will be utilised to minimise discomfort.

NP samples will be collected, stored and transported in line with World Health Organization (WHO) guidelines[1] using sterile nylon flocked swabs. Following collection, swab will be placed immediately into 1000 µL Skim Milk Tryptone Glucose Glycerol Broth (STGGB). The samples will be kept chilled until transportation to the Pasteur Institute, Ho Chi Minh City, Vietnam (Pasteur). On arrival at Pasteur two aliquots will be removed, and the aliquots and original sample will be frozen at ≤ -70°C, within 8 hours of collection. NP swabs collected at 18 and 24 months of age (NPs c and d) will be shipped to the Murdoch Children’s Research Institute, Melbourne, Australia (MCRI) on dry ice and stored at ≤ -70°C prior to analysis.

Blood samples will be collected using a butterfly needle and vacutainer, or, if the infant's veins are difficult to palpate, using a syringe attached to a 23G Surflo winged infusion set or through a finger prick using an appropriate lancet. The volume of blood collected is: 2 mL at 2-3 months of age or for ELISA samples when two samples are collected 7 days apart; 7.5 mL for B cell assays; and 3.5 mL at other time points. 2 mL and 3.5 mL blood samples will be collected into gel vacutainer tubes and kept chilled until transportation to Pasteur in a transport cooler box, unless specific laboratory tests require alternative collection methods. On arrival at the laboratory the sera will be centrifuged in a fridge centrifuge then divided into up to four aliquots, stored in micro-tubes and frozen at ≤ -70°C prior to analysis. 7.5 mL blood samples will be collected into sodium heparin vacutainer tubes and transported to Pasteur at room temperature the same day. On arrival at Pasteur plasma and peripheral blood mononuclear cells (PBMCs) will be separated from each heparinized blood sample by density gradient centrifugation. Plasma will be divided into up to four aliquots and stored at ≤ -70°C prior to analysis. PBMCs will be counted and at least $10x10^6$ cells/mL will be used for B cell assays where indicated, and the remainder counted for viability using the trypan blue exclusion method. PBMCs will then be cryopreserved at ≤ -70°C in aliquots containing 8-$10x10^6$ cells/mL and stored until shipment to MCRI for further analysis.

2 Laboratory evaluations of nasopharyngeal swabs

Traditional microbiology will be used to analyse the NP swabs collected at 6 and 12 months of age (NPs a and b), and qPCR and microarray will be used to analyse the NP swabs collected at 18 and 24 months of age (NPs c and d). Traditional microbiology (the culturing of NP swabs and the identification and typing of *S. pneumoniae*) will be done at Pasteur, under guidance from the microbiology teams at Menzies School of Health Research, Darwin, Australia (Menzies) and MCRI. qPCR and microarray analysis will be performed at MCRI.

2.1 Traditional culture methods

Traditional culture methods were consistent with WHO guidelines.[1] Prior to analysis, batches of swabs will be removed from -70°C storage and thawed on ice. Once thawed, swabs will be vortexed for 10 seconds (sec) and 50 µL of each sample inoculated onto horse blood agar + colistin + nalidixic acid plates (horse blood CNA plates). Plates will be incubated overnight (18-24 hours) at 37°C in 5% CO_2. Identification of *S. pneumoniae* will primarily be based on colonial morphology (typically flat with a dimple 1-3 mm in size), α-haemolysis and the optochin test. One colony of the dominant morphology will be selected, along with an example of each morphologically distinct colony type. Colonies will be sub-cultured onto horse blood agar (HBA) to obtain pure isolates.

Serotyping of pneumococcal isolates will be performed by latex agglutination, with Quellung confirmation as required. Latex agglutination identifies pneumococcal serotypes by using sensitised latex particles.[2] The pneumococcal culture suspension is mixed with 10 µL of sensitised latex particles (produced in-house[3] from antisera obtained from the Statens Serum Institut) on clear glass slides and rotated for 2 minutes (min). A positive test is indicated by aggregation of latex particles and clearing of the suspension. Firstly, the isolate is screened with antisera pools and then with specific serogroup/type/factor reagents as required to determine the final serotype. Quellung serotyping will be used to resolve any inconclusive results from latex agglutination serotyping. Quellung uses antisera to determine the serogroup and serotype (including factor testing) of the pneumococcal isolate.[4] Pneumococcal culture (1 µL) is dotted onto a glass slide and mixed with 1 µL of antisera (Statens Serum Institute). Each drop is covered with a coverslip and examined by microscopy. A positive reaction is indicated by the appearance of capsular swelling. Any pneumococci that are non-typeable will be tested using PCR targeting the *lytA* gene to confirm species identification.[5]

2.2 qPCR and microarray

Each sample will be pelleted by centrifugation for 10 min at 6,000 x *g*. DNA will then be extracted from a 100 µL aliquot of STGGB, using a QIAcube HT (Qiagen) instrument and a QIAamp 96 DNA QIAcube HT Kit (Qiagen) with an initial pre-lysis step. The pre-lysis step includes a 30 min incubation at 37°C with a lysis buffer (20 mM Tris/HCl, 2 mM EDTA, 1% v/v Triton, 20 mg/mL lysozyme, 2 mg/mL RNase A, and 0.075 mg/mL mutanolysin), followed by a 30 min incubation at 56°C with 20 µL of Proteinase K and 200 µL of Buffer AL (Qiagen). qPCR targeting the *lytA* gene will be used to quantify pneumococcal density,[6] by reference to a standard curve from a dilution series of isolate genomic DNA (5 µL per well). Samples that are qPCR positive (cycle threshold (Ct) <35) or equivocal (CT 35-40) will be cultured on HBA containing 5 µg/mL gentamicin (gHBA, Oxoid). Samples that have α-haemolytic colonies will have growth harvested from the culture plates and DNA extracted on a QIAcube HT instrument (Qiagen) as described previously. Molecular serotyping will be conducted by microarray using the extracted DNA and Senti-SPv1.5 microarrays (BUGS Bioscience), with analysis using a custom web-based software.[7] Serotype-specific density will be calculated by multiplication of qPCR data (overall pneumococcal density) and microarray data (relative abundance of serotype(s)).

3 Laboratory evaluations of blood samples

All blood samples will be analysed by ELISA to measure serotype-specific anti-pneumococcal IgG antibody concentrations. A subset of blood samples from 12m and 12m+28d will also be analysed by OPA to measure functional serotype-specific anti-pneumococcal IgG, and a subset of blood samples from 12m, 12m+7d, 12m+28d and 24m will be analysed by ELISPOT assay to determine the memory B cell responses. ELISAs will be performed at Pasteur, under guidance from the immunology team at MCRI; OPAs will be performed at MCRI; and B cell assays will be performed at the Pasteur laboratory, with final reading of the plates performed at MCRI.

3.1 ELISAs

Serotype-specific anti-pneumococcal IgG will be measured for the serotypes in PCV13 using a previously published modified WHO ELISA method[8] and the new international reference serum, 007sp (FDA/CBER).[9] In brief, microtitre wells are coated with pneumococcal polysaccharide diluted in phosphate buffered saline (PBS). To neutralise non-specific antibodies, the reference serum 007sp, three controls and infant serum samples will be absorbed by overnight incubation in diluent containing cell wall polysaccharide and serotype 22F. Samples, controls and standard are loaded to the pre-coated plates and the assay is developed using HRP conjugated anti-human IgG. Detection is completed using a TMB (3,3_,5,5_-tetramethylbenzidine) substrate solution and the reaction stopped with 1M phosphoric acid. A high, medium, and low control serum will be included on each plate to assess assay performance and inter-assay variation. Results will be reported in µg/ml of serotype-specific IgG.

3.2 OPAs

OPA provides a measure of the opsonophagocytic and killing activity of anti- pneumococcal antibodies. Functional serotype-specific IgG will be measured for all serotypes in PCV13 using a multiplexed opsonophagocytic assay (MOPA),[10] a modification of the standardised single OPA.[11] Serial dilutions of heat inactivated infant sera are incubated with cultured HL-60 phagocytic cells (ATCC), rabbit Complement (Pel-Freez) and a mix of cultured antibiotic resistant *Streptococcus pneumoniae*. After 45 min, the serial dilutions are plated to selective Todd-Hewitt broth with Yeast Extract (THYE) agar plates. At 24 hours, the number of colonies per dilution is measured using a ProtoCol 3 colony counter. A control serum sample, a Complement control (no serum) and a bacterial control (no Complement) are included in each assay. Results are recorded as an opsonic index (OI), which is the reciprocal of the serum dilution with at least 50% killing when compared to the average growth in complement control wells. An OI ≥8 is considered a positive response. Negative results are recorded as OI=4.

3.3 B cell assays

The number of circulating memory B cells to pneumococcal serotypes will be determined by ELISPOT assay using a previously published method.[12] In brief, PBMCs are re-suspended in RPMI Foetal Calf Serum (FCS) at a concentration of $2x10^6$ cells/mL and 100µL added to each well of the culture plate containing an antigen cocktail (Staphylococcus aureus Cowan strain – Pansorbin cells (SAC; 1:5000), 2.5µg/mL CpG and 83ng/mL pokeweed mitogen). Plates are incubated at 37°C with 5% CO_2 and 95% humidity for 5 days. At day 5, cells are harvested and washed and the cell pellet re-suspended in 1mL RPMI-FCS and counted by trypan blue. Cells are then made up to a final concentration of $2x10^6$ cells/mL, seeded onto ELISPOT plates coated with anti-IgG (10µg/mL), tetanus toxoid (5µg/mL), diphtheria toxoid (10µg/mL) or pneumococcal polysaccharides conjugated to methylated human serum albumin (10-20µg/mL) and incubated overnight. Bound IgG is detected with an alkaline phosphatase-conjugated IgG and cells are counted using an automated ELISPOT reader and software. The total number of IgG-secreting antibody-forming cells (AFCs) is used as the positive control and 1,000 IgG AFCs/10^6 cultured PBMCs is the lower cut-off for inclusion in the analysis.

4 References

1. Satzke C, Turner P, Virolainen-Julkunen A, et al. Standard method for detecting upper respiratory carriage of *Streptococcus pneumoniae*: updated recommendations from the World Health Organization Pneumococcal Carriage Working Group. *Vaccine* 2013;32:165-79. doi:10.1016/j.vaccine.2013.08.062.
2. Porter BD, Ortika BD, Satzke C. Capsular Serotyping of *Streptococcus pneumoniae* by latex agglutination. *Journal of visualized experiments : JoVE* 2014:51747. doi:10.3791/51747.
3. Ortika BD, Habib M, Dunne EM, et al. Production of latex agglutination reagents for pneumococcal serotyping. *BMC Res Notes* 2013;6:49. doi:10.1186/1756-0500-6-49.
4. Habib M, Porter BD, Satzke C. Capsular serotyping of *Streptococcus pneumoniae* using the Quellung reaction. *Journal of visualized experiments : JoVE* 2014:e51208. doi:10.3791/51208.
5. World Health Organization. Laboratory methods for the diagnosis of meningitis caused by *Neisseria meningitidis, Streptococcus pneumoniae, and Haemophilus influenzae*: WHO manual. 2nd ed, 2011. WHO/IVB.11.09. Available at: https://apps.who.int/iris/handle/10665/70765
6. Satzke C, Dunne EM, Choummanivong M, et al. Pneumococcal carriage in vaccine-eligible children and unvaccinated infants in Lao PDR two years following the introduction of the 13-valent pneumococcal conjugate vaccine. *Vaccine* 2019;37:296-305. doi:10.1016/j.vaccine.2018.10.077.
7. Newton R, Hinds J, Wernisch L. Empirical Bayesian models for analysing molecular serotyping microarrays. *BMC Bioinformatics* 2011;12:88. doi:10.1186/1471-2105-12-88.
8. Balloch A, Licciardi PV, Leach A, et al. Results from an inter-laboratory comparison of pneumococcal serotype-specific IgG measurement and critical parameters that affect assay performance. *Vaccine* 2010;28:1333-40. doi:10.1016/j.vaccine.2009.11.011.
9. Goldblatt D, Plikaytis BD, Akkoyunlu M, et al. Establishment of a new human pneumococcal standard reference serum, 007sp. *Clin Vaccine Immunol* 2011;18:1728-36. doi:10.1128/CVI.05252-11.
10. Burton RL, Nahm MH. Development of a fourfold multiplexed opsonophagocytosis assay for pneumococcal antibodies against additional serotypes and discovery of serological subtypes in *Streptococcus pneumoniae* serotype 20. *Clin Vaccine Immunol* 2012;19:835-41. doi:10.1128/CVI.00086-12.
11. Rose CE, Romero-Steiner S, Burton RL, et al. Multilaboratory comparison of *Streptococcus pneumoniae* opsonophagocytic killing assays and their level of agreement for the determination of functional antibody activity in human reference sera. *Clin Vaccine Immunol* 2011;18:135-42. doi:10.1128/CVI.00370-10.
12. Licciardi PV, Toh ZQ, Clutterbuck EA, et al. No long-term evidence of hyporesponsiveness after use of pneumococcal conjugate vaccine in children previously immunized with pneumococcal polysaccharide vaccine. *The Journal of allergy and clinical immunology* 2016;137:1772-79 e11. doi:10.1016/j.jaci.2015.12.1303.

APPENDIX 3

Murdoch Childrens Research Institute

Information statement and consent form

PART I: INFORMATION STATEMENT

**Research Project Title:**	**Vietnam Pneumococcal Trial II**
**Principal Researcher:**	**Research Partners:**
Edward Kim Mulholland, MD<br>Nguyen Vu Thuong, MD	Murdoch Childrens Research Institute, Melbourne, Australia<br>Pasteur Institute of Ho Chi Minh City, Ho Chi Minh City, Vietnam
**HREC Project Number:**	36027

Thank you for taking the time to read this **Parent/Guardian Information Statement and Consent Form**. We would like to invite your child to participate in a research project that is explained below.

This document is 5 pages long. Please make sure you have all the pages.

What is an Information Statement?

These pages tell you about the research project. It explains to you clearly and openly all the steps and procedures of the project. The information is to help you decide whether or not you would like your child to take part in the research. Please read this Information Statement carefully.

Before you decide if you want your child to take part or not, you can ask us any questions you have about the project. You may want to talk about the project with your family, friends or health care worker.

Important things you need to know

- It is your choice whether or not your child can take part in the research. You do not have to agree if you do not want to
- If you decide you do not want your child to take part, it will not affect the treatment and care your child receives at the CHC clinic.

If you would like your child to take part in the research project, please sign the consent form at the end of this information statement. By signing the consent form you are telling us that you:

- understand what you have read
- had a chance to ask questions and received satisfactory answers
- consent to your child taking part in the project

We will give you a copy of this information and consent form to keep.

Temple B, et al. BMJ Open 2021; 11:e056505. doi: 10.1136/bmjopen-2021-056505

Why are we doing the study?

Pneumonia is a common problem in Vietnam and throughout the developing world. In the developing world it is the leading cause of death in children under 5 years of age. A number of germs cause pneumonia but the most common germ is a bacteria called pneumococcus. Pneumococcus can also cause ear infections as well as other more severe diseases like meningitis (infection around the brain). This germ normally lives in the nose of humans and is spread from person to person by touching or sneezing. There are more than 90 types of this germ but only some types cause serious infections in young children.

Pneumococcal vaccines protect against infection with pneumococcus. There are two licensed pneumococcal vaccines. These are used in the United States and many countries in Europe. Unfortunately the costs of these vaccines are very high, so not all countries in the world can afford them. We are doing this study to find the best ways to protect babies from this germ and also to make it cheaper for countries like Vietnam to afford to buy the vaccine.

We hope that up to 2500 babies will take part in this study.

What does the study involve?

Consent: We will explain what is involved in the study and ask some questions about your baby's health. If you agree to join the study we will ask you to sign a consent form. After this, a study doctor will perform a health check of your baby to make sure your baby is healthy to take part.

Enrolment: Study doctors will examine all children to ensure that participants have no pre-existing health conditions that make them not eligible to take part in the study. To be enrolled in the study a child must:

- Be aged between 2 months and 2 months 2 weeks;
- Have been born at a gestation greater than 36 weeks in an uncomplicated pregnancy;
- Live within approximately 30 minutes from the study clinic and anticipate residing locally for the next 22 months;
- Parent / legal guardian has signed a consent form to participate in the research

Length of study: If you and your baby take part, you will need to come to this commune health centre for between seven and ten visits over 22 months. We will remind you when you need to come.

Questionnaire: At the start of the study you will be asked some questions about your family and your baby's health. These are to help us understand how the vaccines work best.

Health checks: Your baby will have a health check at each study visit. Your baby will have a more detailed health check at the 6 month and 12 month visits, involving physical and developmental assessments.

Vaccinations: There are five different vaccine groups in this study. Like rolling a dice your baby will be allocated to one of the five groups. Your baby will get one or two doses of pneumococcal vaccine, at the ages shown in the table below.

**Group** (number of participants)	**2 months**	**12 months**	**24 months**
**V** (400)		PCV10	
**W** (400)		PCV13	
**X** (400)	PCV10	PCV10	
**Y** (400)	PCV13	PCV13	
**Z** (900)			PCV10

Groups V and W receive a single dose at 12 months; X and Y receive 2 doses at 2 and 12 months and group Z does not receive PCV until 24 months of age.

PCV13 (*Prevnar-13*) covers 13 types of the pneumococcal germ and PCV10 (*Synflorix*) covers ten types of the pneumococcal germ.

Your baby will also get four doses of *Infanrix-hexa* 6-1, an infant vaccine that covers all the diseases that are covered by the standard vaccines used in Vietnam (diphtheria, tetanus, pertussis, hepatitis B, polio virus and *Haemophilus influenzae* type B). Note: Participants will receive the measles vaccine, two doses of Japanese Encephalitis vaccine and a dose of measles-rubella vaccine at their local Commune Health Centre, as per the routine EPI practice.

Nose swabs: Up to four nose swabs will be taken during the study, at 6, 12, 18 and 24 months of age. The nose swabs are to see if the vaccine will help stop the spread of the pneumococcus from child to child. This will involve putting a cotton wool swab (like a cotton bud) into your baby's nose for a couple of seconds. This may make your baby sneeze and possibly cry briefly – it tickles quite a lot, but doesn't really hurt.

Blood tests: Up to three blood tests will be taken during the study, at 12 months, 12 months plus 1 or 4 weeks, and at 2-3 months or at 24 months of age. The volume of blood taken will be 2ml at 2 months of age, 3.5ml at 3 months of age and 3.5ml or 7.5ml at other ages. The blood tests are to check the response to the vaccines. Blood will be taken by staff from Children's Hospital Number 1 or 2. If you like we can put local anaesthetic cream on your baby's skin before taking the blood test so that it doesn't hurt as much.

Hospitalisation: If your baby becomes unwell during the study, we may need to look at your child's medical records. If your baby is admitted to hospital with respiratory symptoms, a chest x-ray and/or a nose swab may be taken. If your baby is admitted to hospital with diarrhoea, a stool sample may be collected.

Benefits of the study

Pneumococcal vaccines are not presently available in the EPI program in Vietnam. By joining the study your baby will have some protection from diseases such as ear infections and pneumonia caused by the commonest pneumococcal germs. In addition children will receive 4 doses of *Infanrix-Hexa*

Are there any risks?

The vaccines we are using are safe and are licensed in many countries (PCV10 is licensed in Vietnam); therefore there is little danger to any child participating in the study. As with all vaccines, your baby may feel some pain or discomfort where the injection is given, and there is a small risk of soreness and redness. Some babies in the study will get one more injection than they would routinely get at 2 months of age. Children will be kept at the study clinic for 30 minutes after each injection to monitor for any unexpected reactions and provide treatment if required. Your baby may feel some pain or discomfort when the blood tests are taken, and there is small risk of bruising, swelling or minor bleeding.

Confidentiality

All information collected in this study will remain confidential and will be used for research purposes only. Your baby will be given an identification number at the start of the study. Any information collected will use this number and will not include your baby's name. All information will be kept secure, stored either in Vietnam or Australia. Information will be stored for at least 15 years after the study finishes.

Some of the samples we collect will be sent to overseas laboratories for tests. These laboratories will not be given your child's name. If you give permission we will keep your baby's blood and nose swab samples indefinitely for other similar tests in the future, either in Vietnam or Australia. This will help us to perform any new pneumococcal test that may be developed in the future.

The results of the study will be published in scientific journals and presented at conferences. There will never be details published that would identify your baby.

Monitors reporting to the donors and state authorities will have access to the research records of your child.

Withdrawal from the Study

You are free to withdraw your baby from the study at any time. This will not affect any of your baby's future health care treatment and there will be no harmful consequences for your baby. If your baby has not had all their pneumococcal vaccines they may not be fully protected against the pneumococcal germs which most commonly affect babies. However, they will still get some protection from any doses of vaccine received.

Compensation

We will pay 200,000VND towards the transport cost for coming to the clinic for each study visit.

What happens if my child is injured or becomes ill during the project?

If your child suffers any injury or complication as a result of this research project contact us as soon as possible. We will help to arrange appropriate medical treatment for your child. If your child becomes ill and requires hospital treatment, if possible they should be taken to Children's Hospital Number 1 or 2. All children participating in the study will be covered by vaccine trial insurance.

Will we be informed of the results when the research project is finished?

We will send you a letter about the overall results at the end of the study.

How is the study funded?

This study is funded by the Bill & Melinda Gates Foundation. The sponsor is the Murdoch Childrens Research Institute, Melbourne, Australia.

Ethical Approval

This study has been approved by the People's Committee of Ho Chi Minh City. This study has also been approved by the Vietnam Ministry of Health Ethics Committee and by The Royal Children's Hospital Melbourne Human Research Ethics Committee. The ethics committees make sure that the study is being done in the best and safest way. If you have any concerns or complaints regarding the conduct of the research project you are invited to contact:

Vietnam Ministry of Health Ethics Committee<br>Phone: 04 62732156	OR	Director, Research Ethics & Governance,<br>The Royal Children's Hospital Melbourne<br>Phone: +61 3 9345 5044

Who should I contact for more information?

Please feel free to contact us if you would like more information about the project or if you need to speak to a member of the research team in an emergency.

If you have any questions regarding the study activities, please phone Dr Tran Phuc Hau: 0904473899

If you have any questions regarding adverse events, please phone study doctors at the site. Contact details are included in the Parent Held Record.

Temple B, *et al. BMJ Open* 2021; 11:e056505. doi: 10.1136/bmjopen-2021-056505

PART II: CONSENT FORM

Participant ID: |__||__||__||__|

Research Project Title: **Vietnam Pneumococcal Trial II**

HREC Project Number: 36027

- I have read, or someone has read to me in a language that I understand, the information statement version listed above and I understand its contents.
- I believe I understand the purpose, extent and possible risks of my child's involvement in this project.
- I voluntarily consent for my child to take part in this research project.
- I have had an opportunity to ask questions and I am satisfied with the answers I have received.
- I understand that this project has been approved by Vietnam Ministry of Health Ethics Committee and The Royal Children's Hospital Melbourne Human Research Ethics Committee and will be carried out in line with the National Statement on Ethical Conduct in Human Research (2007) – including all updates.
- I understand I will receive a copy of this Information Statement and Consent Form.

CONSENT

☐ I do	☐ I do not	agree for my baby to take part in this study

USE OF SAMPLES

☐ I do	☐ I do not	consent to the storage of my child's unused blood/NP samples for future work in the same general area of research that has obtained ethics committee approval

______________________ Gender: Male / Female Date of birth: ______________

Child's Name

______________________ ______________________ ______________

Parent/Guardian Name Parent/Guardian Signature Date

Time: _ _ : _ _

____

Relationship to Child

If illiterate: *A literate witness must sign (if possible, this person should be selected by the participant and should have no connection to the research team).*

I have witnessed the accurate reading of the consent form to the parent of the potential participant, and the individual has had the opportunity to ask questions. I confirm that the individual has given consent freely.

______________________ ______________________ ______________

Witness Name Witness Signature Date

Declaration by researcher: I have explained the project to the parent/guardian who has signed above, and believe that they understand the purpose, extent and possible risks of their child's involvement in this project.

______________________ ______________________ ______________

Research Team Member Name Research Team Member Signature Date

Note: All parties signing the Consent Form must date their own signature.
