# Supplementary material for: Simplified 0+1 and 1+1 pneumococcal vaccine schedules in Ho Chi Minh City, Vietnam: protocol for a randomised controlled trial
Source: BMJ Open. 2021 Nov 29;11(11):e056505. doi: 10.1136/bmjopen-2021-056505 (PMC8634020; doi:10.1136/bmjopen-2021-056505)
Supplement: Author's manuscript [file bmjopen-2021-056505.draft_revisions.pdf]

BMJ Open is committed to open peer review. As part of this commitment we make the peer review history of every article we publish publicly available.

When an article is published we post the peer reviewers' comments and the authors' responses online. We also post the versions of the paper that were used during peer review. These are the versions that the peer review comments apply to.

The versions of the paper that follow are the versions that were submitted during the peer review process. They are not the versions of record or the final published versions. They should not be cited or distributed as the published version of this manuscript.

BMJ Open is an open access journal and the full, final, typeset and author-corrected version of record of the manuscript is available on our site with no access controls, subscription charges or pay-per-view fees (http://bmjopen.bmj.com).

If you have any questions on BMJ Open's open peer review process please email info.bmjopen@bmj.com

BMJ Open

A randomised controlled trial of simplified 0+1 and 1+1 pneumococcal vaccine schedules in Ho Chi Minh City, Vietnam

Journal:	*BMJ Open*
Manuscript ID	bmjopen-2021-056505
Article Type:	Protocol
Date Submitted by the Author:	17-Aug-2021

Complete List of Authors:	Temple, Beth; Menzies School of Health Research, Global and Tropical Health Division; Murdoch Childrens Research Institute<br>Tran, Hau; Pasteur Institute Ho Chi Minh City, Department for Disease Control and Prevention<br>Dai, Vo Thi Trang; Pasteur Institute of Ho Chi Minh City, Department of Microbiology and Immunology<br>Bright, Kathryn; Murdoch Childrens Research Institute<br>Uyen, Doan ; Pasteur Institute of Ho Chi Minh City, Department of Disease Control and Prevention<br>Balloch, Anne; Murdoch Childrens Research Institute<br>Licciardi, Paul; Murdoch Childrens Research Institute; University of Melbourne, Department of Paediatrics<br>Nguyen, Cattram; Murdoch Childrens Research Institute; University of Melbourne, Department of Paediatrics<br>Satzke, Catherine; Murdoch Childrens Research Institute; University of Melbourne, Department of Paediatrics<br>Smith-Vaughan, Heidi; Menzies School of Health Research, Child Health Division<br>Vu Nguyen, Thuong ; Pasteur Institute of Ho Chi Minh City, Department of Disease Control and Prevention<br>Muholland , Kim; Murdoch Childrens Research Institute; London School of Hygiene & Tropical Medicine

Keywords:	Paediatric infectious disease & immunisation < PAEDIATRICS, MICROBIOLOGY, IMMUNOLOGY, Clinical trials < THERAPEUTICS

SCHOLARONE™
Manuscripts

I, the Submitting Author has the right to grant and does grant on behalf of all authors of the Work (as defined in the below author licence), an exclusive licence and/or a non-exclusive licence for contributions from authors who are: i) UK Crown employees; ii) where BMJ has agreed a CC-BY licence shall apply, and/or iii) in accordance with the terms applicable for US Federal Government officers or employees acting as part of their official duties; on a worldwide, perpetual, irrevocable, royalty-free basis to BMJ Publishing Group Ltd ("BMJ") its licensees and where the relevant Journal is co-owned by BMJ to the co-owners of the Journal, to publish the Work in this journal and any other BMJ products and to exploit all rights, as set out in our licence.

The Submitting Author accepts and understands that any supply made under these terms is made by BMJ to the Submitting Author unless you are acting as an employee on behalf of your employer or a postgraduate student of an affiliated institution which is paying any applicable article publishing charge ("APC") for Open Access articles. Where the Submitting Author wishes to make the Work available on an Open Access basis (and intends to pay the relevant APC), the terms of reuse of such Open Access shall be governed by a Creative Commons licence – details of these licences and which Creative Commons licence will apply to this Work are set out in our licence referred to above.

Other than as permitted in any relevant BMJ Author's Self Archiving Policies, I confirm this Work has not been accepted for publication elsewhere, is not being considered for publication elsewhere and does not duplicate material already published. I confirm all authors consent to publication of this Work and authorise the granting of this licence.

For peer review only

Title
A randomised controlled trial of simplified 0+1 and 1+1 pneumococcal vaccine schedules in Ho Chi Minh City, Vietnam

Corresponding author
Beth Temple
Menzies School of Health Research
PO Box 41096
Casuarina NT 0811
Australia
+61 8 8946 8600
beth.temple@menzies.edu.au

Co-authors

Beth TEMPLE[1, 2, 3]
Hau Phuc TRAN[4]
Vo Thi Trang DAI[5]
Kathryn BRIGHT[2]
Doan Y UYEN[4]
Anne BALLOCH[2]
Paul Vincent LICCIARDI[2, 6]
Cattram Duong NGUYEN[2, 6]
Catherine SATZKE[2, 6, 7]
Heidi SMITH-VAUGHAN[8]
Thuong Vu NGUYEN[4]
Kim MULHOLLAND[2, 3]

[1]Global and Tropical Health Division, Menzies School of Health Research, Charles Darwin University, Darwin, NT, Australia
[2]Infection and Immunity, Murdoch Children's Research Institute, Melbourne, VIC, Australia
[3]Epidemiology and Population Health, London School of Hygiene & Tropical Medicine, London, UK
[4]Department of Disease Control and Prevention, Pasteur Institute of Ho Chi Minh City, Ho Chi Minh City, Vietnam
[5]Department of Microbiology and Immunology, Pasteur Institute of Ho Chi Minh City, Ho Chi Minh City, Vietnam
[6]Department of Paediatrics, University of Melbourne, Melbourne, VIC, Australia
[7]Department of Microbiology and Immunology, The University of Melbourne at the Peter Doherty Institute for Infection and Immunity, Melbourne, VIC, Australia
[8]Child Health Division, Menzies School of Health Research, Charles Darwin University, Darwin, NT, Australia

Word count: 3975 words

For peer review only

ABSTRACT

Introduction: Reduced-dose schedules offer a more efficient and affordable way to utilise pneumococcal conjugate vaccines (PCVs). Such schedules rely primarily on the maintenance of herd protection. The Vietnam Pneumococcal Trial II (VPT-II) will evaluate reduced-dose schedules of PCV10 and PCV13. Schedules will be compared in relation to their effect on nasopharyngeal carriage and immunogenicity.
Methods and analysis: VPT-II is a single-blind randomised controlled trial of 2500 infants recruited at 2 months of age to one of five groups: four intervention groups that receive PCV10 in a 0+1 or 1+1 schedule or PCV13 in a 0+1 or 1+1 schedule; and a control group (that receives a single dose of PCV10 at 24 months). Participants are followed up to 24 months of age. The primary outcome is vaccine-type pneumococcal carriage at 24 months of age.
Ethics and dissemination: Ethical approval has been obtained from the Human Research Ethics Committee of the Royal Children's Hospital Melbourne and the Vietnam Ministry of Health Ethics Committee. The results, interpretation and conclusions will be presented to parents and guardians, at national and international conferences, and published in peer-reviewed open access journals.
Trial registration details: ClinicalTrials.gov NCT03098628

Strengths and limitations of this study

- There is considerable interest in reduced-dose pneumococcal conjugate vaccine (PCV) schedules as a strategy to maintain herd protection and to make PCVs more affordable
- The primary outcome in this study is pneumococcal carriage, providing a measure of the likely ability of the PCV schedules being evaluated to generate herd protection
- This study also includes a full range of immunological outcome measures, encompassing antibody responses (by ELISA), functional antibody responses (by OPA) and memory B cell responses
- This study utilises both PCV10 and PCV13, allowing a direct comparison of these two vaccines

For peer review only

For peer review only - http://bmjopen.bmj.com/site/about/guidelines.xhtml

ADMINISTRATIVE INFORMATON

Title

A randomised controlled trial of simplified 0+1 and 1+1 pneumococcal vaccine schedules in Ho Chi Minh City, Vietnam

Trial registration

ClinicalTrials.gov: NCT03098628

Trial registration - data set

Data category	Information
Primary registry and trial identifying number	ClinicalTrials.gov NCT03098628
Date of registration in primary registry	1 March 2017
Secondary identifying numbers	HREC36027
Source(s) of monetary or material support	Bill & Melinda Gates Foundation
Primary sponsor	Murdoch Children's Research Institute, Australia
Contact for public queries	Professor Kim Mulholland kim.mulholland@lshtm.ac.uk
Contact for scientific queries	Professor Kim Mulholland kim.mulholland@lshtm.ac.uk
Public title	A randomised controlled trial of simplified 0+1 and 1+1 pneumococcal vaccine schedules in Ho Chi Minh City, Vietnam
Scientific title	Trial of simplified pneumococcal vaccination in Vietnam II (VPT-II): the herd immunity approach
Countries of recruitment	Vietnam
Health condition(s) or problem(s) studied	Pneumococcal vaccination responses
Intervention(s)	Active Comparator V: PCV10 administered at 12 months of age (0+1 PCV10)
	Active Comparator W: PCV13 administered at 12 months of age (0+1 PCV13)
	Active Comparator X: PCV10 administered at 2 and 12 months of age (1+1 PCV10)
	Active Comparator Y: PCV13 administered at 12 and 2 months of age (1+1 PCV13)
	Control Z: PCV10 administered at end of trial (24 months)
Key inclusion and exclusion criteria	Inclusion criteria:<br>• aged between 2 months and 2 months plus 2 weeks<br>• no significant maternal or perinatal history<br>• born at or after 36 weeks gestation<br>• written and signed informed consent from parent/legal guardian<br>• lives within approximately 30 minutes of the commune health centre

For peer review only - http://bmjopen.bmj.com/site/about/guidelines.xhtml

	• family anticipates living in the study area for the next 22 months
	Exclusion criteria:<br>• known allergy to any component of the vaccine<br>• allergic reaction or anaphylactic reaction to any previous vaccine<br>• known immunodeficiency disorder<br>• known HIV-infected mother<br>• known thrombocytopenia or coagulation disorder<br>• administration or planned administration of any immunoglobulin or blood product since birth<br>• severe birth defect requiring ongoing medical care<br>• chronic or progressive disease; seizure disorder<br>• history of severe illness<br>• receipt of any 2 month vaccines through the EPI program<br>• family plans on giving the infant *Quinvaxem* (DTP-Hib-HBV)
Study type	Interventional, randomised, parallel group, open label phase II/III trial. Outcome assessors (laboratory) blinded. Purpose: prevention.
Enrolment period	8 March 2017 – 11 June 2020
Sample size	Target: 2500<br>Number enrolled: 2501
Recruitment status	Active, not recruiting
Primary outcome	Vaccine-type (VT) pneumococcal carriage at 24 months of age
Key secondary outcomes	• VT pneumococcal carriage at 6, 12 and 18 months of age<br>• Non-VT pneumococcal carriage at 6, 12, 18 and 24 months of age<br>• Carriage of any pneumococcal serotype at 6, 12, 18 and 24 months of age<br>• Serotype-specific IgG antibody concentrations post-2-month dose, pre-12-month dose, post-12-month dose of PCV and at 24 months of age<br>• Serotype-specific opsonophagocytic indices pre- and post-12-month dose of PCV<br>• Serotype-specific memory B cell numbers pre- and post-12-month dose of PCV and at 24 months of age
Ethics Review	Approved by the Human Research Ethics Committee of the Royal Children's Hospital Melbourne and the Vietnam Ministry of Health Ethics Committee

For peer review only

Protocol version

Protocol version 5.0 dated 8 February 2018

Revision chronology
Original: Version 3.2, 11 October 2016.
First amendment: Version 4.0, 16 May 2017. Main reason for amendment: minor clarifications requested by the Vietnam Ministry of Health, along with a change to a final version number
Second amendment: Version 5.0, 8 February 2018. Main reason for amendment: to remove references to JEV and measles-rubella (MR) vaccine, as for logistical reasons these are to be administered through the CHCs and not as part of the study

Roles and responsibilities

Sponsor contact information
Trial Sponsor: Murdoch Children's Research Institute, Royal Children's Hospital, Flemington Road, Parkville, Victoria 3052, Australia
Telephone: +61 3 8341 6200
Contact name: Professor Kim Mulholland

Other Institutions
Menzies School of Health Research, Darwin, Australia
Role: oversight of traditional microbiology (culture methods)

Family Health International (FHI360), Ho Chi Minh City, Vietnam
Role: external clinical trial monitoring

Bill & Melinda Gates Foundation
Role: funding source
The funder does not have any role in the trial conduct, trial management, laboratory tests, or data analyses.

For peer review only

INTRODUCTION

6a Background and rationale

Streptococcus pneumoniae (the pneumococcus) causes significant morbidity and mortality in children under five years of age.[1] There are two infant pneumococcal conjugate vaccines (PCVs) currently in use, PCV10 (*Synflorix*, GlaxoSmithKline) and PCV13 (*Prevnar-13*, Pfizer). A third PCV, *Pneumosil* (10-valent, Serum Institute of India), received World Health Organization (WHO) pre-qualification in December 2019. Both PCV10 and PCV13 are available through the Advanced Market Commitment (AMC) mechanism, a vaccine purchase process developed by Gavi to support vaccine introduction into low and middle-income countries (LMICs). Countries that have introduced PCVs with Gavi support are rapidly approaching the time when they will have to pay most, if not all, of the price of the vaccine, necessitating simpler, less expensive ways of using PCVs.

Introduction of PCV has been associated with dramatic reductions in pneumococcal disease.[2-4] The benefits of vaccination are not only seen amongst vaccinated individuals (direct protection), but also in the wider population (indirect herd protection) through reduced nasopharyngeal (NP) carriage and transmission of vaccine type (VT) pneumococci.[5] The manufacturers recommend a 3+1 schedule (a three-dose primary series with a booster), but WHO currently recommends a three-dose schedule (either 3+0, a three-dose primary series without a booster, or 2+1, a two-dose primary series with a booster).[6] There is evidence to suggest that the number of doses could be further reduced with schedules designed to maintain herd protection. The UK recently became the first country to move to a 1+1 reduced-dose schedule, based on favourable post-booster immunogenicity compared with a 2+1 schedule.[7]

In a 1+1 schedule, the first dose is likely to confer some protection to the recipient, and importantly provides priming for the later booster dose. A single dose of PCV7 showed 73% effectiveness during a period of vaccine shortage in the US,[8] and a single dose of PCV13 in the UK showed 60% effectiveness for the additional serotypes not included in PCV7.[9] A single dose of PCV in infancy also generates a measurable and significant immune response,[10-13] and provides better priming for a booster dose than multiple doses in infancy for some serotypes.[7, 14] The purpose of the second dose in a 1+1 schedule is maintenance of herd protection. The timing of this dose should consider maximising individual protection of the

For peer review only - http://bmjopen.bmj.com/site/about/guidelines.xhtml

recipient (through earlier administration) and optimising protection against carriage (through later administration); 12 months of age offers a balance between these two factors. A further simplified schedule involves a single dose at around 12 months of age, with no primary immunization (a 0+1 schedule). The implication is that a single dose will be sufficient to maintain pre-existing herd protection and control the potential re-emergence of vaccine types, while recognising the reduced individual protection during the first year of life.

Reduced-dose (0+1 or 1+1) schedules could be implemented in countries with established PCV programs, or they could be introduced as a primary course in their own right (in conjunction with a comprehensive catch-up campaign). Such simplified regimens present an opportunity to use existing PCVs more efficiently, and to make PCVs more affordable. The Vietnam Pneumococcal Trial II (VPT-II) will evaluate both PCV10 and PCV13 in a 0+1 schedule at 12 months of age and a 1+1 schedule at 2 and 12 months of age in relation to their effect on NP carriage and immunogenicity during the first two years of life.

6b Explanation for choice of comparators

PCV is not part of the routine infant vaccination program in Vietnam. PCV10 is available on the private market but is not widely used. Inclusion of a control group that receives no infant doses of PCV is therefore justified. Control group participants receive a single dose of PCV10 at 24 months of age.

7 Objectives

The overall objective of this trial is to fully evaluate the microbiological and immunological effects of 0+1 and 1+1 schedules for both PCV10 and PCV13.

Co-primary objectives

- To evaluate the effect of a 0+1 PCV schedule on NP carriage during the first two years of life, comparing a) PCV10-vaccinated and b) PCV13-vaccinated participants with unvaccinated controls
- To evaluate the effect of a 1+1 PCV schedule on NP carriage during the first two years of life, comparing a) PCV10-vaccinated and b) PCV13-vaccinated participants with unvaccinated controls

Secondary objectives

For peer review only - http://bmjopen.bmj.com/site/about/guidelines.xhtml

- To evaluate the immunogenicity of a 0+1 schedule of PCV10 or PCV13
- To evaluate the immunogenicity of a 1+1 schedule of PCV10 or PCV13
- To determine whether the response to a dose of PCV10 or PCV13 at 12 months of age is enhanced by a dose at 2 months of age (i.e. 0+1 vs. 1+1)
- To investigate the kinetics of the immune response to PCV vaccination at 12 months of age, comparing antibody levels and memory B cell responses 7- and 28-days post-vaccination
- To examine the serotype profile of transferred maternal pneumococcal antibodies at 2 months of age
- To directly compare PCV10 and PCV13 in terms of immune responses and effect on NP carriage

For peer review only

8 Trial design

VPT-II is a single-blind, open-label, randomised controlled phase II/III clinical trial to investigate simplified PCV schedules that are focussed primarily on the generation of herd immunity. Participants are randomised to one of five groups: Group V receives a 0+1 PCV10 schedule, Group W receives a 0+1 PCV13 schedule, Group X receives a 1+1 PCV10 schedule, Group Y receives a 1+1 PCV13 schedule, and Group Z is a control group (that receives a single dose of PCV10 at 24 months of age).

METHODS

9 Study setting

The trial is conducted in three districts within Ho Chi Minh City, Vietnam (Districts 4, 7 and 8). Districts are divided into communes, each of which has a health centre that provides preventive health services including EPI vaccines, along with primary healthcare services. The trial is conducted in one commune health centre in each district, with participants drawn from the surrounding communes within that district.

10 Eligibility criteria

Inclusion criteria

For peer review only - http://bmjopen.bmj.com/site/about/guidelines.xhtml

In order to be eligible, subjects must meet all of the following criteria: aged between 2 months and 2 months plus 2 weeks, no significant maternal or perinatal history, born at or after 36 weeks gestation, written and signed informed consent from parent/legal guardian, lives within approximately 30 minutes of the commune health centre, and family anticipates living in the study area for the next 22 months.

Exclusion criteria

Subjects meeting any of the following criteria will not be eligible to participate: known allergy to any component of the vaccine, allergic reaction or anaphylactic reaction to any previous vaccine, known immunodeficiency disorder, known HIV-infected mother, known thrombocytopenia or coagulation disorder, administration or planned administration of any immunoglobulin or blood product since birth, severe birth defect requiring ongoing medical care, chronic or progressive disease, seizure disorder, history of severe illness, receipt of any 2 month vaccines through the EPI program, or family plans on giving the infant *Quinvaxem* (DTP-Hib-HBV).

11 Interventions

The interventions are PCV10 and PCV13. There are four intervention groups, which receive: PCV10 at 12 months of age (0+1 PCV10, Group V), PCV13 at 12 months of age (0+1 PCV13, Group W), PCV10 at 2 and 12 months of age (1+1 PCV10, Group X), or PCV13 at 2 and 12 months of age (1+1 PCV13, Group Y). Control group participants (Group Z) receive a single dose of PCV10 at 24 months of age. PCV is administered by intramuscular injection into the anterolateral thigh in children less than 18 months old and in the deltoid muscle of the arm in children aged 18 months and over. All vaccinations are performed by nurses specifically trained in infant vaccine administration. Single-dose vials of PCV10 and single-dose pre-filled syringes of PCV13 are used.

11b Criteria for discontinuing or modifying allocated interventions

There is no modification of doses for participants in this study. If a participant has an allergic or anaphylactic response to vaccination, they will be withdrawn from the study. Participants may also be withdrawn voluntarily by the parent/legal guardian at any time, or by the study staff if they refuse any further study procedures or develop any of the exclusion criteria during the course of the study.

For peer review only - http://bmjopen.bmj.com/site/about/guidelines.xhtml

11c Strategies to improve and monitor adherence

Scheduled visit dates are noted on a health record card kept by the parent. If a participant does not attend a scheduled visit, a reminder phone call is made from the study clinic. If the participant cannot be contacted directly, their local Commune Health Centre is contacted for further follow up by phone or by home visit.

11d Relevant concomitant care

Participants also receive four doses of *Infanrix-hexa*, which is only available on the private market, instead of the routine EPI vaccine *Quinvaxem*. Other vaccinations are permitted in this study with a two-week interval from study vaccines, with the exception of *Quinvaxem*. Other medications are also permitted, with the exception of immunosuppressive medication and medications listed as contraindicated to the study vaccines.

12 Outcomes

Primary outcome measure

The primary outcome measure to address each of the co-primary objectives is carriage of VT pneumococci, defined as carriage of serotypes 1, 4, 5, 6B, 7F, 9V, 14, 18C, 19F or 23F for the PCV10 groups, or carriage of serotypes 1, 3, 4, 5, 6A, 6B, 7F, 9V, 14, 18C, 19A, 19F or 23F for the PCV13 groups. The VT pneumococcal carriage prevalence (defined as the proportion of participants positive for VT pneumococcal carriage) will be determined at 24 months of age (primary endpoint), and also at 6, 12 and 18 months of age (secondary endpoints). Traditional culture methods (colonial morphology, α-haemolysis and the optochin test) and serotyping by latex agglutination/Quellung, with *lytA* PCR confirmation of nonencapsulated isolates, will be the main methods used to analyse NP swabs collected at 6 and 12 months of age. Quantitative real-time PCR (qPCR) targeting the *lytA* gene[15] and serotyping by microarray[16] will be the main methods used to analyse NP swabs collected at 18 and 24 months of age.

Secondary microbiological outcome measures

- Carriage of any pneumococcal serotype at 6, 12, 18 and 24 months of age
- Non-VT pneumococcal carriage at 6, 12, 18 and 24 months of age
- Serotype-specific pneumococcal carriage at 6, 12, 18 and 24 months of age
- Density of pneumococcal carriage (overall, VT, non-VT and serotype-specific) at 18 and 24 months of age

For peer review only - http://bmjopen.bmj.com/site/about/guidelines.xhtml

Secondary immunological outcome measures

- Serotype-specific IgG antibody concentrations for all PCV13 serotypes, measured by ELISA[17] from all blood samples
- Opsonisation indices (OI) for all PCV13 serotypes, measured by opsonophagocytic assay (OPA)[18] for 50 participants per intervention group (Groups V-Y) pre- and four weeks post-12 months dose of PCV
- Polysaccharide specific memory B cells for serotypes 1, 5, 6A, 6B, 14, 19A and 23F, enumerated by ELISPOT[19] for 50 participants per intervention group (Groups V-Y) pre-, 7 days post- and 28 days post-12 months dose of PCV and at 24 months of age

An overview of the procedures for collection, transportation and laboratory analyses of the blood and NP samples can be found in Appendix 1.

13 Participant timeline

Participants are enrolled at 2 months of age and followed up to 24 months of age (Table 1a). Group V-Y participants provide NP swabs at 6 and 12 months of age, and all participants provide NP swabs at 18 and 24 months of age for analysis of the NP carriage outcomes. A subset of 200 participants per group will be included in the immunology sub-study and provide three (Groups V-Y) or one (Group Z) blood sample(s) over the course of the trial for analysis of vaccine responses (Table 1b).

Table 1a: Schedule of enrolment, interventions and assessments

**Age (months)**	**2m**	**3m**	**4m**	**6m**	**12m**	**18m**	**24m**
ENROLMENT:							
Informed consent	✓						
Eligibility assessment	✓						
Allocation	✓						
INTERVENTIONS:							
PCV10	X				V, X		Z
PCV13	Y				W, Y		
Infanrix-hexa	✓	✓	✓			✓	
ASSESSMENTS:							
Demographics	✓						
Household characteristics	✓						
Nasopharyngeal swab				V-Y	V-Y	✓	✓
General health	✓	✓	✓	✓	✓	✓	✓

✓ indicates applies to all groups (V-Z), otherwise group(s) specified.

For peer review only - http://bmjopen.bmj.com/site/about/guidelines.xhtml

Table 1b: Schedule of blood samples for immunology sub-study (subset of Groups V-Z)

Age (months)	2m	3m	12m	Post-12m[1]	24m
Sub-group a			V-Y[2]	V-Y	V-Y
Sample volume			7.5ml	2ml	3.5ml
Assays			ELISA, B cell	ELISA	ELISA
Sub-group b	V	W-Y	V-Y	V-Y	
Sample volume	2ml	2ml	2ml	7.5ml	
Assays	ELISA	ELISA	ELISA	ELISA, B cell	
Sub-group c			V-Y	V-Y	V-Y
Sample volume			3.5ml	7.5ml	3.5ml
Assays			ELISA	ELISA, B cell	ELISA
Sub-group d			V-Y	V-Y	V-Y
Sample volume			3.5ml	3.5ml	7.5ml
Assays			ELISA, OPA	ELISA, OPA	ELISA, B cell
Sub-group Z-I					Z
Sample volume					3.5ml
Assays					ELISA

[1] The post-12m blood sample is collected at 12 months plus 7 days in sub-groups a and b and at 12 months plus 28 days in sub-groups c and d. [2] 3.5ml sample for ELISA only in group W.

14 Sample size

The target sample size is 2500 with an allocation ratio of 4:4:4:4:9. This allocation ratio provides the greatest power for a total sample size of 2500 and results in target group sizes of 400 for each of the four different infant vaccination schedules (Groups V-Y) and 900 for the control group (Group Z). Sample size calculations are based on the primary outcome of effect on VT pneumococcal carriage at 24 months of age. For each of the four vaccination schedules compared with the unvaccinated control group, the proposed sample size provides 82% power to detect a 40% reduction in the vaccinated group, with a two-sided type I error rate of 5%. The sample size calculations assume 15% VT pneumococcal carriage among controls, based on data from our previous pneumococcal vaccine trial in Ho Chi Minh City,[20] and 10% loss-to-follow-up.

15 Recruitment

Participants are recruited from infants born in the study communes during the enrolment period. Potential participants are identified from commune health centre birth records and are visited by commune health centre staff when they are

For peer review only - http://bmjopen.bmj.com/site/about/guidelines.xhtml

approximately six weeks old. Verbal and written information about the trial is provided in Vietnamese and those interested in participating are referred to the study clinic when the infant is approximately two months old. At this time, written informed consent is obtained (Appendix 2), after which a study nurse/doctor examines the infant to ensure that all the eligibility criteria are met. Recruitment rates will be monitored on a monthly basis and meetings held with study staff and commune health centre staff to discuss any significant declines in recruitment rates.

16 Allocation

The allocation ratio of Groups V, W, X, Y and Z is 4:4:4:4:9. The first 200 participants enrolled into each of Groups V-Y, and a randomly selected 200 of the first 450 participants concurrently enrolled into Group Z, will go into the immunology sub-study. These participants (with the exception of Group Z) will be further randomised into one of four sub-groups (a, b, c or d) in an equal allocation ratio (Table 1b). Randomisation will be conducted by a database manager in Australia, using a computer-generated list of random numbers in a block randomisation scheme stratified by district. The group allocation, and sub-group allocation where relevant, is contained within a sealed envelope at the study clinic. Participants are assigned to a study group by a study doctor, using the next available envelope. The envelopes are prepared and sealed in the study office in Vietnam, by staff with no involvement in the recruitment process.

17 Blinding

All outcome measures are laboratory-based. Laboratory staff are blinded to the study group allocation. Laboratory samples are labelled with a unique ID number, which does not identify the study group. Given the different timing of the vaccination schedules in the different groups, the study nurses, vaccine administrators and participants will not be blinded to the study group allocation.

18 Data collection methods

Data collected at the clinic are documented by dedicated, trained study staff using standardised forms. Blood samples and NP swabs are collected by staff specifically trained in the collection of samples from infants, and the volume of blood and swab quality are recorded. Laboratory data generated in both Vietnam and Australia are entered directly into dedicated databases in the laboratories by the laboratory staff and sent periodically to the data management team in Australia.

For peer review only - http://bmjopen.bmj.com/site/about/guidelines.xhtml

Retention

Appointments are documented on a parent-held health record card and a reminder phone call made the week before the scheduled visit. Missed visits are re-booked by phone and participants who miss a study visit will continue to be followed up for both sample collection and vaccine administration where possible. Participants receive a small payment at each visit towards transport costs.

19 Data management

Data collected at the clinic are entered on-site into a secure, web-based electronic data capture system, REDCap.[21] All entered data are monitored against the source documents for accuracy and completeness, and a series of data checks are performed on a regular basis to identify potential errors in the data. An audit trail of corrections and changes to the data is stored within the REDCap database. Immunology results are double-entered in a Microsoft Excel spreadsheet. NP culture results are entered in a Microsoft Access database and qPCR and microarray results are exported from SentiNET into Microsoft Excel or REDCap databases. The data collection forms and laboratory results are linked at the time of analysis.

20 Statistical methods

20a Analysis of primary and secondary outcomes

The primary outcome of VT carriage will be presented as a proportion (prevalence). VT carriage prevalence in each of the vaccinated groups (PCV10-type carriage for Groups V and X and PCV13-type carriage for Groups W and Y) will be compared with unvaccinated controls. Prevalence ratios (ratio of prevalence in each vaccinated group to prevalence in unvaccinated controls) with 95% confidence intervals will be calculated, and groups compared using chi-squared tests (two-sided p-values reported). The primary endpoint is 24 months of age, with secondary endpoints of 6, 12, and 18 months of age. At 6 and 12 months of age, time points at which no swabs were collected from Group Z, swabs from the 0+1 groups (Groups V and W combined) will form the unvaccinated comparator.

Immunological outcomes will be summarised in terms of: geometric mean concentrations (GMCs) and the proportion with protective antibody levels ($\geq$0.35μg/mL)[22] with 95% CIs for serotype-specific IgG data, geometric mean opsonisation indices (GMOIs) and the proportion with an OI $\geq$8[23] with 95% CIs for

For peer review only - http://bmjopen.bmj.com/site/about/guidelines.xhtml

OPA data, and the mean number of antibody secreting cells per 10^6 PBMCs for polysaccharide-specific memory B cell data. GMC and GMOI ratios and risk differences with 95% CIs will also be calculated. Means will be compared using T-tests and proportions compared using Fisher's Exact tests.

20c Populations of analysis

Analysis is planned on an intention-to-treat population (ITT), with all participants to be analysed in the group to which they were randomised. Withdrawn participants may not contribute data at all time points as blood and NP samples may not be collected after their withdrawal.

21 Data monitoring

21a Data monitoring committee

Safety oversight is under the direction of an independent Data Safety and Monitoring Board (DSMB), in accordance with a DSMB Charter kept in the trial office. The DSMB will consist of at least three members, including two physicians and one biostatistician. The DSMB will meet periodically to review aggregate and individual participant data related to safety, data integrity and overall conduct of the trial, including a detailed review of all Serious Adverse Events (SAEs).

21b Interim analyses and stopping guidelines

No interim analyses are planned. Statistical rules will not be used to halt study enrolment or vaccine administration. Stopping guidelines are based on safety. An extraordinary meeting of the DSMB will be called in the event that serious safety issues emerge, to provide recommendations regarding termination of the trial. A final decision to terminate rests with the Principal Investigators and the Sponsor.

22 Harms

Data on SAEs will be collected throughout the duration of the main study, with parents asked about hospitalisations and significant signs and symptoms at each study visit and through a regular review of hospital records. All SAEs will be recorded on the standard Vietnam Ministry of Health reporting form and reported to the Principal Investigators and the Ethics Committees. Participants will be kept under observation for 30 minutes following vaccine administration to monitor for any adverse reactions, and adverse events that may contraindicate further vaccinations will be assessed following all vaccination visits. Reactogenicity will be assessed following all doses of PCV using parent held diary cards.

For peer review only

Auditing

External site monitoring will be provided by Family Health International (FHI360), to independently assess protocol and GCP compliance. The frequency of monitoring visits and the level of detail of the monitoring will be documented in a contract between FHI360 and the sponsor. Monitoring visits will start prior to enrolment of the first participant and continue through to study-close-out.

Patient and public involvement

Patients were not involved in the development, design, recruitment or conduct of the study. Community consultation took place at the district level during the design phase, as well as discussion and approval of the design from the district health centres, the city Department of Health, and the People's Committee of Ho Chi Minh City. Participants will be informed of the overall study results by email or by post, with addresses collected at the final study visit.

ETHICS AND DISSEMINATION

24 Research ethics approval

The protocol and the Informed Consent Form (ICF) have approval from the Institutional Review Board at the Pasteur Institute of Ho Chi Minh City, the Vietnam Ministry of Health Ethical Review Committee for Bio-medical Research and the Human Research Ethics Committee of the Royal Children's Hospital, Melbourne. Both Ethics Committees receive annual reports on the trial progress, for continuing approval of the trial.

25 Protocol amendments

Any modifications to the protocol that may impact on the conduct of the study will be documented in a formal protocol amendment and approved by both Ethics Committees prior to implementation of the changes. The modified protocol will be given a new version number and date. The Ethics Committees will also be notified of any minor corrections/clarifications or administrative changes to the protocol, which will be documented in a protocol amendment letter. Significant protocol changes will also be updated in the ClinicalTrials.gov record.

26 Consent

26a Obtaining consent

For peer review only

The consent process is undertaken by specifically trained study staff. The study staff go through the ICF, translated into Vietnamese, in detail with the potential participant's parent/legal guardian. Study staff then discuss the trial further and answer any questions that may arise. Written informed consent is required prior to enrolment of the infant into the study and is provided by the parent/legal guardian as the participants are too young to provide consent themselves. A copy of the ICF will be given to the parent/legal guardian for their records.

26b Ancillary studies

Specific consent for the indefinite storage of blood and NP samples for future research related to the trial will be obtained from the parent/legal guardian and recorded on the ICF. Any future research will undergo ethical review. Any samples for which indefinite storage is not consented to will be destroyed at the close of the study.

27 Confidentiality

All study-related information will be stored securely and held in strict confidence. All documents kept at the study clinics are stored in locked cabinets. The REDCap database is password protected. All documents maintained centrally are stored in the trial office, which is kept locked. The laboratory samples and electronic laboratory data are coded by participant ID number and do not contain names. Access to participants' information will be granted to FHI360 for monitoring purposes, and to the Ethics Committees or DSMB if required.

28 Declaration of Interests

KM, CS and CN are investigators on a collaborative study on PCV impact on adult pneumonia funded by Pfizer.

29 Access to data

The final dataset will be under the custody of the trial sponsor, MCRI. The Principal Investigator, trial manager and trial statistician will have access to the full anonymised final dataset.

30 Ancillary and post-trial care

Participants are advised to come to the study clinic for ancillary care, or to Children's Hospital Number 1 or Children’s Hospital Number 2 in Ho Chi Minh City, where they

For peer review only

will not be charged for treatment and services. All participants are covered by clinical trials insurance for trial related harms.

31 Dissemination policy

31a Plans

Following completion of the trial, the results will be submitted for publication in peer-reviewed journals and presented at relevant international conferences. The results will be disseminated regardless of the magnitude or direction of effect. This research is undertaken as a collaboration between MCRI and the Pasteur Institute of Ho Chi Minh City. Either party must obtain the prior approval of the other party in advance of submitting a manuscript for publication, and such approval will not be unreasonably withheld.

31b Authorship

A publication subcommittee will consider all proposed publications, with the final decision on content and authorship resting with the Principal Investigator. The role of each author will be published in line with journal requirements. Group authors may be used where appropriate. There are no plans for the use of professional writers.

31c Reproducible research

Data will be made publicly available in accordance with the rules set out by the Bill & Melinda Gates Foundation.

For peer review only

AUTHORS' CONTRIBUTIONS

BT wrote the first draft of this manuscript and drafted the trial protocol with input from HPT, KB and DYU. HPT also advised on the study design and oversaw the approval processes in Vietnam. VTTD, AB, PVL, CS and HSV were involved in the laboratory-related aspects of the design and protocol development. CDN advised on the study design and statistical aspects of the trial. TVN was involved in the design and establishment of the trial and had overall responsibility for its conduct in Vietnam as the site Principal Investigator. KM conceived the study, provided oversight for all aspects of the design and implementation, and had overall responsibility for the trial as Principal Investigator. All authors contributed to refinement of the trial protocol and reviewed and approved this manuscript.

ACKNOWLEDGEMENTS AND FUNDING STATEMENT

We thank the study participants and their families and the study staff. We acknowledge the contributions of Phan Trong Lan, Sophie La Vincente, Helen Thomson and Thi Que Huong Vu (deceased). This work is supported by the Bill & Melinda Gates Foundation (grant number OPP-1116833/INV-008627). We also acknowledge the Victorian Government's Operational Infrastructure Support Program.

COMPETING INTERESTS STATEMENT

All authors receive salary support from grants from the Bill & Melinda Gates Foundation. KM, CS and CN are investigators on a collaborative study on PCV impact on adult pneumonia funded by Pfizer. None of the authors have any other competing interests to declare.

APPENDICES

Appendix 1 – Sample collection and processing

Appendix 2 - Informed Consent Form. These materials were translated into Vietnamese.

For peer review only

References

1. Collaborators GBDLRI. Estimates of the global, regional, and national morbidity, mortality, and aetiologies of lower respiratory infections in 195 countries, 1990-2016: a systematic analysis for the Global Burden of Disease Study 2016. *The Lancet. Infectious diseases* 2018;18:1191-210. doi:10.1016/S1473-3099(18)30310-4.
2. Myint TT, Madhava H, Balmer P, et al. The impact of 7-valent pneumococcal conjugate vaccine on invasive pneumococcal disease: a literature review. *Advances in therapy* 2013;30:127-51. doi:10.1007/s12325-013-0007-6.
3. Conklin L, Loo JD, Kirk J, et al. Systematic review of the effect of pneumococcal conjugate vaccine dosing schedules on vaccine-type invasive pneumococcal disease among young children. *Pediatr Infect Dis J* 2014;33 Suppl 2:S109-18. doi:10.1097/INF.0000000000000078.
4. Loo JD, Conklin L, Fleming-Dutra KE, et al. Systematic review of the effect of pneumococcal conjugate vaccine dosing schedules on prevention of pneumonia. *Pediatr Infect Dis J* 2014;33 Suppl 2:S140-51. doi:10.1097/INF.0000000000000082.
5. Shiri T, Datta S, Madan J, et al. Indirect effects of childhood pneumococcal conjugate vaccination on invasive pneumococcal disease: a systematic review and meta-analysis. *The Lancet. Global health* 2017;5:e51-e59. doi:10.1016/S2214-109X(16)30306-0.
6. Pneumococcal conjugate vaccines in infants and children under 5 years of age: WHO position paper - February 2019. *Wkly Epidemiol Rec* 2019;94:85-104.
7. Goldblatt D, Southern J, Andrews NJ, et al. Pneumococcal conjugate vaccine 13 delivered as one primary and one booster dose (1 + 1) compared with two primary doses and a booster (2 + 1) in UK infants: a multicentre, parallel group randomised controlled trial. *The Lancet. Infectious diseases* 2017. doi:10.1016/S1473-3099(17)30654-0.
8. Whitney CG, Pilishvili T, Farley MM, et al. Effectiveness of seven-valent pneumococcal conjugate vaccine against invasive pneumococcal disease: a matched case-control study. *Lancet* 2006;368:1495-502. doi:10.1016/S0140-6736(06)69637-2.
9. Andrews NJ, Waight PA, Burbidge P, et al. Serotype-specific effectiveness and correlates of protection for the 13-valent pneumococcal conjugate vaccine: a postlicensure indirect cohort study. *The Lancet. Infectious diseases* 2014;14:839-46. doi:10.1016/S1473-3099(14)70822-9.
10. Huebner RE, Mbelle N, Forrest B, et al. Immunogenicity after one, two or three doses and impact on the antibody response to coadministered antigens of a nonavalent pneumococcal conjugate vaccine in infants of Soweto, South Africa. *Pediatr Infect Dis J* 2002;21:1004-7. doi:10.1097/01.inf.0000036012.37665.fe.
11. Russell FM, Balloch A, Tang ML, et al. Immunogenicity following one, two, or three doses of the 7-valent pneumococcal conjugate vaccine. *Vaccine* 2009;27:5685-91. doi:10.1016/j.vaccine.2009.06.098.
12. Russell FM, Carapetis JR, Burton RL, et al. Opsonophagocytic activity following a reduced dose 7-valent pneumococcal conjugate vaccine infant primary series and 23-valent pneumococcal polysaccharide vaccine at 12 months of age. *Vaccine* 2011;29:535-44. doi:10.1016/j.vaccine.2010.10.046.
13. Licciardi PV, Temple B, Dai VTT, et al. Immunogenicity of alternative ten-valent pneumococcal conjugate vaccine schedules in infants in Ho Chi Minh City, Vietnam: results from a single-blind, parallel-group, open-label, randomised, controlled trial. *The Lancet. Infectious diseases* 2021. doi:10.1016/S1473-3099(20)30775-1.

For peer review only

14. Russell FM, Licciardi PV, Balloch A, et al. Safety and immunogenicity of the 23-valent pneumococcal polysaccharide vaccine at 12 months of age, following one, two, or three doses of the 7-valent pneumococcal conjugate vaccine in infancy. *Vaccine* 2010;28:3086-94. doi:10.1016/j.vaccine.2010.02.065.
15. Satzke C, Dunne EM, Choummanivong M, et al. Pneumococcal carriage in vaccine-eligible children and unvaccinated infants in Lao PDR two years following the introduction of the 13-valent pneumococcal conjugate vaccine. *Vaccine* 2019;37:296-305. doi:10.1016/j.vaccine.2018.10.077.
16. Dunne EM, Satzke C, Ratu FT, et al. Effect of ten-valent pneumococcal conjugate vaccine introduction on pneumococcal carriage in Fiji: results from four annual cross-sectional carriage surveys. *The Lancet. Global health* 2018;6:e1375-e85. doi:10.1016/S2214-109X(18)30383-8.
17. Balloch A, Licciardi PV, Leach A, et al. Results from an inter-laboratory comparison of pneumococcal serotype-specific IgG measurement and critical parameters that affect assay performance. *Vaccine* 2010;28:1333-40. doi:10.1016/j.vaccine.2009.11.011.
18. Balloch A, Roalfe L, Ekstrom N, et al. Interlaboratory Comparison of the Pneumococcal Multiplex Opsonophagocytic Assays and Their Level of Agreement for Determination of Antibody Function in Pediatric Sera. *mSphere* 2018;3. doi:10.1128/mSphere.00070-18.
19. Licciardi PV, Toh ZQ, Clutterbuck EA, et al. No long-term evidence of hyporesponsiveness after use of pneumococcal conjugate vaccine in children previously immunized with pneumococcal polysaccharide vaccine. *The Journal of allergy and clinical immunology* 2016;137:1772-79 e11. doi:10.1016/j.jaci.2015.12.1303.
20. Temple B, Nation ML, Dai VTT, et al. Effect of a 2+1 schedule of ten-valent versus 13-valent pneumococcal conjugate vaccine on pneumococcal carriage: Results from a randomised controlled trial in Vietnam. *Vaccine* 2021;39:2303-10. doi:10.1016/j.vaccine.2021.02.043.
21. Harris PA, Taylor R, Thielke R, et al. Research electronic data capture (REDCap)--a metadata-driven methodology and workflow process for providing translational research informatics support. *Journal of biomedical informatics* 2009;42:377-81. doi:10.1016/j.jbi.2008.08.010.
22. WHO Expert Committee on Biological Standardization. WHO Technical Report Series 927. Geneva: World Health Organisation, 2005.
23. Burton RL, Nahm MH. Development and validation of a fourfold multiplexed opsonization assay (MOPA4) for pneumococcal antibodies. *Clin Vaccine Immunol* 2006;13:1004-9. doi:13/9/1004 [pii]
10.1128/CVI.00112-06.

For peer review only

APPENDICES

Table of contents

APPENDIX 1: Sample Collection and Processing 2

1 Sample Collection 2
2 Laboratory evaluations of nasopharyngeal swabs 3
2.1 Traditional culture methods 3
2.2 qPCR and microarray 4
3 Laboratory evaluations of blood samples 4
3.1 ELISAs 4
3.2 OPAs 5
3.3 B cell assays 5
4 References 6

APPENDIX 2: Information Statement and Consent Form 7

PART I: Information Statement 7
PART II: Consent Form 11

For peer review only

APPENDIX 1

Sample collection and processing

1 Sample Collection

Participants will provide four (Groups V-Y) or two (Group Z) nasopharyngeal (NP) swabs over the course of the trial. Additionally, participants in the immunology sub-study will provide three (Groups V-Y) or one (Group Z) venous blood sample(s) over the course of the trial. A minimum of two members of the study team will be present during the collection of NP swabs and blood samples, to ensure no injury is caused by an infant's sudden movement. Distraction techniques will be utilised to minimise discomfort.

NP samples will be collected, stored and transported in line with World Health Organization (WHO) guidelines[1] using sterile nylon flocked swabs. Following collection, swab will be placed immediately into 1000 µL Skim Milk Tryptone Glucose Glycerol Broth (STGGB). The samples will be kept chilled until transportation to the Pasteur Institute, Ho Chi Minh City, Vietnam (Pasteur). On arrival at Pasteur two aliquots will be removed, and the aliquots and original sample will be frozen at ≤ -70°C, within 8 hours of collection. NP swabs collected at 18 and 24 months of age (NPs c and d) will be shipped to the Murdoch Children's Research Institute, Melbourne, Australia (MCRI) on dry ice and stored at ≤ -70°C prior to analysis.

Blood samples will be collected using a butterfly needle and vacutainer, or, if the infant's veins are difficult to palpate, using a syringe attached to a 23G Surflo winged infusion set or through a finger prick using an appropriate lancet. The volume of blood collected is: 2 mL at 2-3 months of age or for ELISA samples when two samples are collected 7 days apart; 7.5 mL for B cell assays; and 3.5 mL at other time points. 2 mL and 3.5 mL blood samples will be collected into gel vacutainer tubes and kept chilled until transportation to Pasteur in a transport cooler box, unless specific laboratory tests require alternative collection methods. On arrival at the laboratory the sera will be centrifuged in a fridge centrifuge then divided into up to four aliquots, stored in micro-tubes and frozen at ≤ -70°C prior to analysis. 7.5 mL blood samples will be collected into sodium heparin vacutainer tubes and transported to Pasteur at room temperature the same day. On arrival at Pasteur plasma and peripheral blood mononuclear cells (PBMCs) will be separated from each heparinized blood sample by density gradient centrifugation. Plasma will be divided into up to four aliquots and stored at ≤ -70°C prior to analysis. PBMCs will be counted and at least $10x10^6$ cells/mL will be used for B cell assays where indicated, and the remainder counted for viability using the trypan blue exclusion method. PBMCs will then be cryopreserved at ≤ -70°C in aliquots containing 8-$10x10^6$ cells/mL and stored until shipment to MCRI for further analysis.

For peer review only

2 Laboratory evaluations of nasopharyngeal swabs

Traditional microbiology will be used to analyse the NP swabs collected at 6 and 12 months of age (NPs a and b), and qPCR and microarray will be used to analyse the NP swabs collected at 18 and 24 months of age (NPs c and d). Traditional microbiology (the culturing of NP swabs and the identification and typing of *S. pneumoniae*) will be done at Pasteur, under guidance from the microbiology teams at Menzies School of Health Research, Darwin, Australia (Menzies) and MCRI. qPCR and microarray analysis will be performed at MCRI.

2.1 Traditional culture methods

Traditional culture methods were consistent with WHO guidelines.[1] Prior to analysis, batches of swabs will be removed from -70°C storage and thawed on ice. Once thawed, swabs will be vortexed for 10 seconds (sec) and 50 μL of each sample inoculated onto horse blood agar + colistin + nalidixic acid plates (horse blood CNA plates). Plates will be incubated overnight (18-24 hours) at 37°C in 5% CO_2. Identification of *S. pneumoniae* will primarily be based on colonial morphology (typically flat with a dimple 1-3 mm in size), α-haemolysis and the optochin test. One colony of the dominant morphology will be selected, along with an example of each morphologically distinct colony type. Colonies will be sub-cultured onto horse blood agar (HBA) to obtain pure isolates.

Serotyping of pneumococcal isolates will be performed by latex agglutination, with Quellung confirmation as required. Latex agglutination identifies pneumococcal serotypes by using sensitised latex particles.[2] The pneumococcal culture suspension is mixed with 10 μL of sensitised latex particles (produced in-house[3] from antisera obtained from the Statens Serum Institut) on clear glass slides and rotated for 2 minutes (min). A positive test is indicated by aggregation of latex particles and clearing of the suspension. Firstly, the isolate is screened with antisera pools and then with specific serogroup/type/factor reagents as required to determine the final serotype. Quellung serotyping will be used to resolve any inconclusive results from latex agglutination serotyping. Quellung uses antisera to determine the serogroup and serotype (including factor testing) of the pneumococcal isolate.[4] Pneumococcal culture (1 μL) is dotted onto a glass slide and mixed with 1 μL of antisera (Statens Serum Institute). Each drop is covered with a coverslip and examined by microscopy. A positive reaction is indicated by the appearance of capsular swelling. Any pneumococci that are non-typeable will be tested using PCR targeting the *lytA* gene to confirm species identification.[5]

For peer review only - http://bmjopen.bmj.com/site/about/guidelines.xhtml

2.2 qPCR and microarray

Each sample will be pelleted by centrifugation for 10 min at 6,000 x *g*. DNA will then be extracted from a 100 µL aliquot of STGGB, using a QIAcube HT (Qiagen) instrument and a QIAamp 96 DNA QIAcube HT Kit (Qiagen) with an initial pre-lysis step. The pre-lysis step includes a 30 min incubation at 37°C with a lysis buffer (20 mM Tris/HCl, 2 mM EDTA, 1% v/v Triton, 20 mg/mL lysozyme, 2 mg/mL RNase A, and 0.075 mg/mL mutanolysin), followed by a 30 min incubation at 56°C with 20 µL of Proteinase K and 200 µL of Buffer AL (Qiagen). qPCR targeting the *lytA* gene will be used to quantify pneumococcal density,[6] by reference to a standard curve from a dilution series of isolate genomic DNA (5 µL per well). Samples that are qPCR positive (cycle threshold (Ct) <35) or equivocal (CT 35-40) will be cultured on HBA containing 5 µg/mL gentamicin (gHBA, Oxoid). Samples that have α-haemolytic colonies will have growth harvested from the culture plates and DNA extracted on a QIAcube HT instrument (Qiagen) as described previously. Molecular serotyping will be conducted by microarray using the extracted DNA and Senti-SPv1.5 microarrays (BUGS Bioscience), with analysis using a custom web-based software.[7] Serotype-specific density will be calculated by multiplication of qPCR data (overall pneumococcal density) and microarray data (relative abundance of serotype(s)).

3 Laboratory evaluations of blood samples

All blood samples will be analysed by ELISA to measure serotype-specific anti-pneumococcal IgG antibody concentrations. A subset of blood samples from 12m and 12m+28d will also be analysed by OPA to measure functional serotype-specific anti-pneumococcal IgG, and a subset of blood samples from 12m, 12m+7d, 12m+28d and 24m will be analysed by ELISPOT assay to determine the memory B cell responses. ELISAs will be performed at Pasteur, under guidance from the immunology team at MCRI; OPAs will be performed at MCRI; and B cell assays will be performed at the Pasteur laboratory, with final reading of the plates performed at MCRI.

3.1 ELISAs

Serotype-specific anti-pneumococcal IgG will be measured for the serotypes in PCV13 using a previously published modified WHO ELISA method[8] and the new international reference serum, 007sp (FDA/CBER).[9] In brief, microtitre wells are coated with pneumococcal polysaccharide diluted in phosphate buffered saline (PBS). To neutralise non-specific antibodies, the reference serum 007sp, three controls and infant serum samples will be absorbed by overnight incubation in diluent containing cell wall polysaccharide and serotype 22F. Samples, controls and standard are loaded to the pre-coated plates and the assay is developed using HRP conjugated anti-human IgG. Detection is completed using a TMB (3,3_,5,5_-tetramethylbenzidine) substrate solution and the reaction stopped with 1M phosphoric acid. A high, medium, and low control serum will be included on each plate to assess assay performance and inter-assay variation. Results will be reported in µg/ml of serotype-specific IgG.

For peer review only

3.2 OPAs

OPA provides a measure of the opsonophagocytic and killing activity of anti- pneumococcal antibodies. Functional serotype-specific IgG will be measured for all serotypes in PCV13 using a multiplexed opsonophagocytic assay (MOPA),[10] a modification of the standardised single OPA.[11] Serial dilutions of heat inactivated infant sera are incubated with cultured HL-60 phagocytic cells (ATCC), rabbit Complement (Pel-Freez) and a mix of cultured antibiotic resistant *Streptococcus pneumoniae*. After 45 min, the serial dilutions are plated to selective Todd-Hewitt broth with Yeast Extract (THYE) agar plates. At 24 hours, the number of colonies per dilution is measured using a ProtoCol 3 colony counter. A control serum sample, a Complement control (no serum) and a bacterial control (no Complement) are included in each assay. Results are recorded as an opsonic index (OI), which is the reciprocal of the serum dilution with at least 50% killing when compared to the average growth in complement control wells. An OI ≥8 is considered a positive response. Negative results are recorded as OI=4.

3.3 B cell assays

The number of circulating memory B cells to pneumococcal serotypes will be determined by ELISPOT assay using a previously published method.[12] In brief, PBMCs are re-suspended in RPMI Foetal Calf Serum (FCS) at a concentration of $2x10^6$ cells/mL and 100µL added to each well of the culture plate containing an antigen cocktail (Staphylococcus aureus Cowan strain – Pansorbin cells (SAC; 1:5000), 2.5µg/mL CpG and 83ng/mL pokeweed mitogen). Plates are incubated at 37^oC with 5% CO_2 and 95% humidity for 5 days. At day 5, cells are harvested and washed and the cell pellet re-suspended in 1mL RPMI-FCS and counted by trypan blue. Cells are then made up to a final concentration of $2x10^6$ cells/mL, seeded onto ELISPOT plates coated with anti-IgG (10µg/mL), tetanus toxoid (5µg/mL), diphtheria toxoid (10µg/mL) or pneumococcal polysaccharides conjugated to methylated human serum albumin (10-20µg/mL) and incubated overnight. Bound IgG is detected with an alkaline phosphatase-conjugated IgG and cells are counted using an automated ELISPOT reader and software. The total number of IgG-secreting antibody-forming cells (AFCs) is used as the positive control and 1,000 IgG AFCs/10^6 cultured PBMCs is the lower cut-off for inclusion in the analysis.

For peer review only

4 References

1. Satzke C, Turner P, Virolainen-Julkunen A, et al. Standard method for detecting upper respiratory carriage of *Streptococcus pneumoniae*: updated recommendations from the World Health Organization Pneumococcal Carriage Working Group. *Vaccine* 2013;32:165-79. doi:10.1016/j.vaccine.2013.08.062.
2. Porter BD, Ortika BD, Satzke C. Capsular Serotyping of *Streptococcus pneumoniae* by latex agglutination. *Journal of visualized experiments : JoVE* 2014:51747. doi:10.3791/51747.
3. Ortika BD, Habib M, Dunne EM, et al. Production of latex agglutination reagents for pneumococcal serotyping. *BMC Res Notes* 2013;6:49. doi:10.1186/1756-0500-6-49.
4. Habib M, Porter BD, Satzke C. Capsular serotyping of *Streptococcus pneumoniae* using the Quellung reaction. *Journal of visualized experiments : JoVE* 2014:e51208. doi:10.3791/51208.
5. World Health Organization. Laboratory methods for the diagnosis of meningitis caused by *Neisseria meningitidis, Streptococcus pneumoniae, and Haemophilus influenzae*: WHO manual. 2nd ed, 2011. WHO/IVB.11.09. Available at: https://apps.who.int/iris/handle/10665/70765
6. Satzke C, Dunne EM, Choummanivong M, et al. Pneumococcal carriage in vaccine-eligible children and unvaccinated infants in Lao PDR two years following the introduction of the 13-valent pneumococcal conjugate vaccine. *Vaccine* 2019;37:296-305. doi:10.1016/j.vaccine.2018.10.077.
7. Newton R, Hinds J, Wernisch L. Empirical Bayesian models for analysing molecular serotyping microarrays. *BMC Bioinformatics* 2011;12:88. doi:10.1186/1471-2105-12-88.
8. Balloch A, Licciardi PV, Leach A, et al. Results from an inter-laboratory comparison of pneumococcal serotype-specific IgG measurement and critical parameters that affect assay performance. *Vaccine* 2010;28:1333-40. doi:10.1016/j.vaccine.2009.11.011.
9. Goldblatt D, Plikaytis BD, Akkoyunlu M, et al. Establishment of a new human pneumococcal standard reference serum, 007sp. *Clin Vaccine Immunol* 2011;18:1728-36. doi:10.1128/CVI.05252-11.
10. Burton RL, Nahm MH. Development of a fourfold multiplexed opsonophagocytosis assay for pneumococcal antibodies against additional serotypes and discovery of serological subtypes in *Streptococcus pneumoniae* serotype 20. *Clin Vaccine Immunol* 2012;19:835-41. doi:10.1128/CVI.00086-12.
11. Rose CE, Romero-Steiner S, Burton RL, et al. Multilaboratory comparison of *Streptococcus pneumoniae* opsonophagocytic killing assays and their level of agreement for the determination of functional antibody activity in human reference sera. *Clin Vaccine Immunol* 2011;18:135-42. doi:10.1128/CVI.00370-10.
12. Licciardi PV, Toh ZQ, Clutterbuck EA, et al. No long-term evidence of hyporesponsiveness after use of pneumococcal conjugate vaccine in children previously immunized with pneumococcal polysaccharide vaccine. *The Journal of allergy and clinical immunology* 2016;137:1772-79 e11. doi:10.1016/j.jaci.2015.12.1303.

For peer review only

7

APPENDIX 2

Information statement and consent form

PART I: INFORMATION STATEMENT

Research Project Title: **Vietnam Pneumococcal Trial II**

Principal Researcher:
Edward Kim Mulholland, MD
Nguyen Vu Thuong, MD

Research Partners:
Murdoch Childrens Research Institute, Melbourne, Australia
Pasteur Institute of Ho Chi Minh City, Ho Chi Minh City, Vietnam

HREC Project Number: 36027

For peer review only

Thank you for taking the time to read this **Parent/Guardian Information Statement and Consent Form**. We would like to invite your child to participate in a research project that is explained below.

This document is 5 pages long. Please make sure you have all the pages.

What is an Information Statement?

These pages tell you about the research project. It explains to you clearly and openly all the steps and procedures of the project. The information is to help you decide whether or not you would like your child to take part in the research. Please read this Information Statement carefully.

Before you decide if you want your child to take part or not, you can ask us any questions you have about the project. You may want to talk about the project with your family, friends or health care worker.

Important things you need to know

- It is your choice whether or not your child can take part in the research. You do not have to agree if you do not want to
- If you decide you do not want your child to take part, it will not affect the treatment and care your child receives at the CHC clinic.

If you would like your child to take part in the research project, please sign the consent form at the end of this information statement. By signing the consent form you are telling us that you:

- understand what you have read
- had a chance to ask questions and received satisfactory answers
- consent to your child taking part in the project

We will give you a copy of this information and consent form to keep.

For peer review only - http://bmjopen.bmj.com/site/about/guidelines.xhtml

Why are we doing the study?

Pneumonia is a common problem in Vietnam and throughout the developing world. In the developing world it is the leading cause of death in children under 5 years of age. A number of germs cause pneumonia but the most common germ is a bacteria called pneumococcus. Pneumococcus can also cause ear infections as well as other more severe diseases like meningitis (infection around the brain). This germ normally lives in the nose of humans and is spread from person to person by touching or sneezing. There are more than 90 types of this germ but only some types cause serious infections in young children.

Pneumococcal vaccines protect against infection with pneumococcus. There are two licensed pneumococcal vaccines. These are used in the United States and many countries in Europe. Unfortunately the costs of these vaccines are very high, so not all countries in the world can afford them. We are doing this study to find the best ways to protect babies from this germ and also to make it cheaper for countries like Vietnam to afford to buy the vaccine.

We hope that up to 2500 babies will take part in this study.

What does the study involve?

Consent: We will explain what is involved in the study and ask some questions about your baby's health. If you agree to join the study we will ask you to sign a consent form. After this, a study doctor will perform a health check of your baby to make sure your baby is healthy to take part.

Enrolment: Study doctors will examine all children to ensure that participants have no pre-existing health conditions that make them not eligible to take part in the study. To be enrolled in the study a child must:

- Be aged between 2 months and 2 months 2 weeks;
- Have been born at a gestation greater than 36 weeks in an uncomplicated pregnancy;
- Live within approximately 30 minutes from the study clinic and anticipate residing locally for the next 22 months;
- Parent / legal guardian has signed a consent form to participate in the research

Length of study: If you and your baby take part, you will need to come to this commune health centre for between seven and ten visits over 22 months. We will remind you when you need to come.

Questionnaire: At the start of the study you will be asked some questions about your family and your baby's health. These are to help us understand how the vaccines work best.

Health checks: Your baby will have a health check at each study visit. Your baby will have a more detailed health check at the 6 month and 12 month visits, involving physical and developmental assessments.

Vaccinations: There are five different vaccine groups in this study. Like rolling a dice your baby will be allocated to one of the five groups. Your baby will get one or two doses of pneumococcal vaccine, at the ages shown in the table below.

**Group** (number of participants)	**2 months**	**12 months**	**24 months**
**V** (400)		PCV10	
**W** (400)		PCV13	
**X** (400)	PCV10	PCV10	
**Y** (400)	PCV13	PCV13	
**Z** (900)			PCV10

Groups V and W receive a single dose at 12 months; X and Y receive 2 doses at 2 and 12 months and group Z does not receive PCV until 24 months of age.

PCV13 (*Prevnar-13*) covers 13 types of the pneumococcal germ and PCV10 (*Synflorix*) covers ten types of the pneumococcal germ.

For peer review only - http://bmjopen.bmj.com/site/about/guidelines.xhtml

Your baby will also get four doses of *Infanrix-hexa* 6-1, an infant vaccine that covers all the diseases that are covered by the standard vaccines used in Vietnam (diphtheria, tetanus, pertussis, hepatitis B, polio virus and *Haemophilus influenzae* type B). Note: Participants will receive the measles vaccine, two doses of Japanese Encephalitis vaccine and a dose of measles-rubella vaccine at their local Commune Health Centre, as per the routine EPI practice.

Nose swabs: Up to four nose swabs will be taken during the study, at 6, 12, 18 and 24 months of age. The nose swabs are to see if the vaccine will help stop the spread of the pneumococcus from child to child. This will involve putting a cotton wool swab (like a cotton bud) into your baby's nose for a couple of seconds. This may make your baby sneeze and possibly cry briefly – it tickles quite a lot, but doesn't really hurt.

Blood tests: Up to three blood tests will be taken during the study, at 12 months, 12 months plus 1 or 4 weeks, and at 2-3 months or at 24 months of age. The volume of blood taken will be 2ml at 2 months of age, 3.5ml at 3 months of age and 3.5ml or 7.5ml at other ages. The blood tests are to check the response to the vaccines. Blood will be taken by staff from Children's Hospital Number 1 or 2. If you like we can put local anaesthetic cream on your baby's skin before taking the blood test so that it doesn't hurt as much.

Hospitalisation: If your baby becomes unwell during the study, we may need to look at your child's medical records. If your baby is admitted to hospital with respiratory symptoms, a chest x-ray and/or a nose swab may be taken. If your baby is admitted to hospital with diarrhoea, a stool sample may be collected.

Benefits of the study

Pneumococcal vaccines are not presently available in the EPI program in Vietnam. By joining the study your baby will have some protection from diseases such as ear infections and pneumonia caused by the commonest pneumococcal germs. In addition children will receive 4 doses of *Infanrix-Hexa*

Are there any risks?

The vaccines we are using are safe and are licensed in many countries (PCV10 is licensed in Vietnam); therefore there is little danger to any child participating in the study. As with all vaccines, your baby may feel some pain or discomfort where the injection is given, and there is a small risk of soreness and redness. Some babies in the study will get one more injection than they would routinely get at 2 months of age. Children will be kept at the study clinic for 30 minutes after each injection to monitor for any unexpected reactions and provide treatment if required. Your baby may feel some pain or discomfort when the blood tests are taken, and there is small risk of bruising, swelling or minor bleeding.

Confidentiality

All information collected in this study will remain confidential and will be used for research purposes only. Your baby will be given an identification number at the start of the study. Any information collected will use this number and will not include your baby's name. All information will be kept secure, stored either in Vietnam or Australia. Information will be stored for at least 15 years after the study finishes.

Some of the samples we collect will be sent to overseas laboratories for tests. These laboratories will not be given your child's name. If you give permission we will keep your baby's blood and nose swab samples indefinitely for other similar tests in the future, either in Vietnam or Australia. This will help us to perform any new pneumococcal test that may be developed in the future.

The results of the study will be published in scientific journals and presented at conferences. There will never be details published that would identify your baby.

Monitors reporting to the donors and state authorities will have access to the research records of your child.

For peer review only - http://bmjopen.bmj.com/site/about/guidelines.xhtml

Withdrawal from the Study

You are free to withdraw your baby from the study at any time. This will not affect any of your baby's future health care treatment and there will be no harmful consequences for your baby. If your baby has not had all their pneumococcal vaccines they may not be fully protected against the pneumococcal germs which most commonly affect babies. However, they will still get some protection from any doses of vaccine received.

Compensation

We will pay 200,000VND towards the transport cost for coming to the clinic for each study visit.

What happens if my child is injured or becomes ill during the project?

If your child suffers any injury or complication as a result of this research project contact us as soon as possible. We will help to arrange appropriate medical treatment for your child. If your child becomes ill and requires hospital treatment, if possible they should be taken to Children's Hospital Number 1 or 2. All children participating in the study will be covered by vaccine trial insurance.

Will we be informed of the results when the research project is finished?

We will send you a letter about the overall results at the end of the study.

How is the study funded?

This study is funded by the Bill & Melinda Gates Foundation. The sponsor is the Murdoch Childrens Research Institute, Melbourne, Australia.

Ethical Approval

This study has been approved by the People's Committee of Ho Chi Minh City. This study has also been approved by the Vietnam Ministry of Health Ethics Committee and by The Royal Children's Hospital Melbourne Human Research Ethics Committee. The ethics committees make sure that the study is being done in the best and safest way. If you have any concerns or complaints regarding the conduct of the research project you are invited to contact:

Vietnam Ministry of Health Ethics Committee<br>Phone: 04 62732156	OR	Director, Research Ethics & Governance,<br>The Royal Children's Hospital Melbourne<br>Phone: +61 3 9345 5044

Who should I contact for more information?

Please feel free to contact us if you would like more information about the project or if you need to speak to a member of the research team in an emergency.

If you have any questions regarding the study activities, please phone Dr Tran Phuc Hau: 0904473899

If you have any questions regarding adverse events, please phone study doctors at the site. Contact details are included in the Parent Held Record.

For peer review only - http://bmjopen.bmj.com/site/about/guidelines.xhtml

PART II: CONSENT FORM

Participant ID: |__||__||__||__|

**Research Project Title:**	**Vietnam Pneumococcal Trial II**
**HREC Project Number:**	36027

- I have read, or someone has read to me in a language that I understand, the information statement version listed above and I understand its contents.
- I believe I understand the purpose, extent and possible risks of my child's involvement in this project.
- I voluntarily consent for my child to take part in this research project.
- I have had an opportunity to ask questions and I am satisfied with the answers I have received.
- I understand that this project has been approved by Vietnam Ministry of Health Ethics Committee and The Royal Children's Hospital Melbourne Human Research Ethics Committee and will be carried out in line with the National Statement on Ethical Conduct in Human Research (2007) – including all updates.
- I understand I will receive a copy of this Information Statement and Consent Form.

CONSENT

☐ I do	☐ I do not	agree for my baby to take part in this study

USE OF SAMPLES

☐ I do	☐ I do not	consent to the storage of my child's unused blood/NP samples for future work in the same general area of research that has obtained ethics committee approval

______________________________ Gender: Male / Female Date of birth: ______________

Child's Name

______________________________ ______________________________ ______________

Parent/Guardian Name Parent/Guardian Signature Date

Time: _ _ : _ _

____

Relationship to Child

If illiterate: *A literate witness must sign (if possible, this person should be selected by the participant and should have no connection to the research team).*

I have witnessed the accurate reading of the consent form to the parent of the potential participant, and the individual has had the opportunity to ask questions. I confirm that the individual has given consent freely.

______________________________ ______________________________ ______________

Witness Name Witness Signature Date

Declaration by researcher: I have explained the project to the parent/guardian who has signed above, and believe that they understand the purpose, extent and possible risks of their child's involvement in this project.

______________________________ ______________________________ ______________

Research Team Member Name Research Team Member Signature Date

Note: All parties signing the Consent Form must date their own signature.

For peer review only - http://bmjopen.bmj.com/site/about/guidelines.xhtml

BMJ Open

Protocol for a randomised controlled trial of simplified 0+1 and 1+1 pneumococcal vaccine schedules in Ho Chi Minh City, Vietnam

Journal:	*BMJ Open*
Manuscript ID	bmjopen-2021-056505.R1
Article Type:	Protocol
Date Submitted by the Author:	26-Oct-2021
Complete List of Authors:	Temple, Beth; Menzies School of Health Research, Charles Darwin University, Global and Tropical Health Division; Murdoch Childrens Research Institute, Infection and Immunity<br>Tran, Hau; Pasteur Institute Ho Chi Minh City, Department for Disease Control and Prevention<br>Dai, Vo Thi Trang; Pasteur Institute of Ho Chi Minh City, Department of Microbiology and Immunology<br>Bright, Kathryn; Murdoch Childrens Research Institute, Infection and Immunity<br>Uyen, Doan ; Pasteur Institute of Ho Chi Minh City, Department of Disease Control and Prevention<br>Balloch, Anne; Murdoch Childrens Research Institute, Infection and Immunity<br>Licciardi, Paul; Murdoch Childrens Research Institute, Infection and Immunity; University of Melbourne, Department of Paediatrics<br>Nguyen, Cattram; Murdoch Childrens Research Institute, Infection and Immunity; University of Melbourne, Department of Paediatrics<br>Satzke, Catherine; Murdoch Childrens Research Institute, Infection and Immunity; University of Melbourne, Department of Paediatrics<br>Smith-Vaughan, Heidi; Menzies School of Health Research, Charles Darwin University, Child Health Division<br>Vu Nguyen, Thuong ; Pasteur Institute of Ho Chi Minh City, Department of Disease Control and Prevention<br>Muholland , Kim; Murdoch Childrens Research Institute, Infection and Immunity; London School of Hygiene & Tropical Medicine, Epidemiology and Population Health
**Primary Subject Heading**:	Global health
Secondary Subject Heading:	Epidemiology
Keywords:	Paediatric infectious disease & immunisation < PAEDIATRICS, MICROBIOLOGY, IMMUNOLOGY, Clinical trials < THERAPEUTICS

For peer review only - http://bmjopen.bmj.com/site/about/guidelines.xhtml

I, the Submitting Author has the right to grant and does grant on behalf of all authors of the Work (as defined in the below author licence), an exclusive licence and/or a non-exclusive licence for contributions from authors who are: i) UK Crown employees; ii) where BMJ has agreed a CC-BY licence shall apply, and/or iii) in accordance with the terms applicable for US Federal Government officers or employees acting as part of their official duties; on a worldwide, perpetual, irrevocable, royalty-free basis to BMJ Publishing Group Ltd ("BMJ") its licensees and where the relevant Journal is co-owned by BMJ to the co-owners of the Journal, to publish the Work in this journal and any other BMJ products and to exploit all rights, as set out in our licence.

The Submitting Author accepts and understands that any supply made under these terms is made by BMJ to the Submitting Author unless you are acting as an employee on behalf of your employer or a postgraduate student of an affiliated institution which is paying any applicable article publishing charge ("APC") for Open Access articles. Where the Submitting Author wishes to make the Work available on an Open Access basis (and intends to pay the relevant APC), the terms of reuse of such Open Access shall be governed by a Creative Commons licence – details of these licences and which Creative Commons licence will apply to this Work are set out in our licence referred to above.

Other than as permitted in any relevant BMJ Author's Self Archiving Policies, I confirm this Work has not been accepted for publication elsewhere, is not being considered for publication elsewhere and does not duplicate material already published. I confirm all authors consent to publication of this Work and authorise the granting of this licence.

For peer review only

Title
Protocol for a randomised controlled trial of simplified 0+1 and 1+1 pneumococcal vaccine schedules in Ho Chi Minh City, Vietnam

Corresponding author
Beth Temple
Menzies School of Health Research
PO Box 41096
Casuarina NT 0811
Australia
+61 8 8946 8600
beth.temple@menzies.edu.au

Co-authors

Beth TEMPLE[1, 2, 3]
Hau Phuc TRAN[4]
Vo Thi Trang DAI[5]
Kathryn BRIGHT[2]
Doan Y UYEN[4]
Anne BALLOCH[2]
Paul Vincent LICCIARDI[2, 6]
Cattram Duong NGUYEN[2, 6]
Catherine SATZKE[2, 6, 7]
Heidi SMITH-VAUGHAN[8]
Thuong Vu NGUYEN[4]
Kim MULHOLLAND[2, 3]

[1]Global and Tropical Health Division, Menzies School of Health Research, Charles Darwin University, Darwin, NT, Australia
[2]Infection and Immunity, Murdoch Children's Research Institute, Melbourne, VIC, Australia
[3]Epidemiology and Population Health, London School of Hygiene & Tropical Medicine, London, UK
[4]Department of Disease Control and Prevention, Pasteur Institute of Ho Chi Minh City, Ho Chi Minh City, Vietnam
[5]Department of Microbiology and Immunology, Pasteur Institute of Ho Chi Minh City, Ho Chi Minh City, Vietnam
[6]Department of Paediatrics, University of Melbourne, Melbourne, VIC, Australia
[7]Department of Microbiology and Immunology, The University of Melbourne at the Peter Doherty Institute for Infection and Immunity, Melbourne, VIC, Australia
[8]Child Health Division, Menzies School of Health Research, Charles Darwin University, Darwin, NT, Australia

Word count: 3975 words

For peer review only

ABSTRACT

Introduction: Reduced-dose schedules offer a more efficient and affordable way to utilise pneumococcal conjugate vaccines (PCVs). Such schedules rely primarily on the maintenance of herd protection. The Vietnam Pneumococcal Trial II (VPT-II) will evaluate reduced-dose schedules of PCV10 and PCV13 utilising an unvaccinated control group. Schedules will be compared in relation to their effect on nasopharyngeal carriage and immunogenicity.
Methods and analysis: VPT-II is a single-blind open-label randomised controlled trial of 2500 infants in three districts of Ho Chi Minh City, Vietnam. Eligible infants have no clinically significant maternal or perinatal history and are born at or after 36 weeks' gestation. Participants are recruited at 2 months of age and randomly assigned (4:4:4:4:9) using block randomisation, stratified by district, to one of five groups: four intervention groups that receive PCV10 in a 0+1 (at 12 months) or 1+1 (at 2 and 12 months) schedule or PCV13 in the same 0+1 or 1+1 schedule; and a control group (that receives a single dose of PCV10 at 24 months). Participants are followed up to 24 months of age. The primary outcome is vaccine-type pneumococcal carriage at 24 months of age. Secondary outcomes are carriage at 6, 12 and 18 months of age and the comparative immunogenicity of the different schedules in terms of antibody responses, functional antibody responses and memory B cell responses.
Ethics and dissemination: Ethical approval has been obtained from the Human Research Ethics Committee of the Royal Children's Hospital Melbourne and the Vietnam Ministry of Health Ethics Committee. The results, interpretation and conclusions will be presented to parents and guardians, at national and international conferences, and published in peer-reviewed open access journals.
Trial registration details: ClinicalTrials.gov NCT03098628

Strengths and limitations of this study

- Conducted in a country without routine PCV use, allowing inclusion of an unvaccinated control group and measurement of the reduction in carriage afforded by the reduced-dose schedules
- Includes 0+1 and 1+1 schedules of both PCV10 and PCV13, allowing a head-to-head comparison of these two vaccines
- Utilises molecular methods to provide a detailed assessment of the effect of reduced-dose schedules on pneumococcal carriage and density at key post-vaccination time points
- Also includes a full range of immunological outcome measures, encompassing antibody responses (by ELISA), functional antibody responses (by OPA) and memory B cell responses
- Does not include an accepted (WHO-recommended) schedule as an additional comparator group

For peer review only

INTRODUCTION

Background and rationale

Streptococcus pneumoniae (the pneumococcus) causes significant morbidity and mortality in children under five years of age.[1] There are two infant pneumococcal conjugate vaccines (PCVs) currently in use, PCV10 (*Synflorix*, GlaxoSmithKline) and PCV13 (*Prevnar-13*, Pfizer). A third PCV, *Pneumosil* (10-valent, Serum Institute of India), received World Health Organization (WHO) pre-qualification in December 2019. Both PCV10 and PCV13 are available through the Advanced Market Commitment (AMC) mechanism, a vaccine purchase process developed by Gavi to support vaccine introduction into low and middle-income countries (LMICs). Under this mechanism, countries pay a gradually increasing share of the cost of their Gavi-supported vaccines. Countries that have introduced PCVs with Gavi support are rapidly approaching the time when they will have to pay most, if not all, of the price of the vaccine, necessitating simpler, less expensive ways of using PCVs.

Introduction of PCV has been associated with dramatic reductions in pneumococcal disease.[2-4] The benefits of vaccination are not only seen amongst vaccinated individuals (direct protection), but also in the wider unvaccinated population (indirect herd protection) through reduced nasopharyngeal (NP) carriage and transmission of vaccine type (VT) pneumococci.[5] The manufacturers recommend a 3+1 schedule (a three-dose primary series with a booster), but WHO currently recommends a three-dose schedule (either 3+0, a three-dose primary series without a booster, or 2+1, a two-dose primary series with a booster).[6] There is evidence to suggest that the number of doses could be further reduced with schedules designed to maintain herd protection. The UK, a country with established herd immunity from a mature PCV programme in a 2+1 schedule, recently became the first country to move to a 1+1 reduced-dose schedule. That decision was based on favourable post-booster immunogenicity compared with a 2+1 schedule, with equivalent or superior antibody levels following a 1+1 schedule for nine of the 13 serotypes in PCV13.[7]

In a 1+1 schedule, the first dose is likely to confer some protection to the recipient, and importantly provides priming for the later booster dose. A single dose of PCV7 showed 73% effectiveness against invasive pneumococcal disease (IPD) during a period of vaccine shortage in the US,[8] and a single dose of PCV13 in the UK showed 60% effectiveness against IPD from the additional serotypes not included in PCV7.[9] A single dose of PCV in infancy also generates a measurable and

For peer review only

significant immune response,[10-13] and is better than multiple doses at priming for a booster dose for some serotypes.[7, 14] The purpose of the second dose in a 1+1 schedule is maintenance of herd protection. The timing of this dose should consider maximising individual protection of the recipient (through earlier administration) and optimising protection against carriage (through later administration); 12 months of age offers a balance between these two factors. In Vietnam, this also coincides with the first routine Japanese Encephalitis Vaccine visit. A further simplified schedule is a 0+1 schedule, involving only the second dose from a 1+1 schedule with no primary immunization. The rationale is that a single dose will be sufficient to maintain pre-existing herd protection and control the potential re-emergence of vaccine types, while recognising the reduced individual protection during the first year of life.

Reduced-dose (0+1 or 1+1) schedules could be implemented in countries with established PCV programs, or they could be introduced as a primary course in their own right (in conjunction with a comprehensive catch-up campaign). Such simplified regimens present an opportunity to use existing PCVs more efficiently, and to make pneumococcal vaccination more affordable. The Vietnam Pneumococcal Trial II (VPT-II) will evaluate both PCV10 and PCV13 in a 0+1 schedule at 12 months of age and a 1+1 schedule at 2 and 12 months of age, in a largely PCV-naïve population utilising an unvaccinated control group, to assess their effect on NP carriage and immunogenicity during the first two years of life.

Explanation for choice of comparators

PCV is not part of the routine infant vaccination program in Vietnam. Inclusion of a control group that receives no infant doses of PCV is therefore justified. PCV13 was not licensed in Vietnam at the time the trial started, and PCV10 was available on the private market but not widely used. Control group participants receive a single dose of PCV10 at 24 months of age in order that all trial participants receive the benefit of pneumococcal vaccination, regardless of group allocation.

Objectives

The overall objective of this trial is to fully evaluate the microbiological and immunological effects of 0+1 and 1+1 schedules for both PCV10 and PCV13.

Co-primary objectives

For peer review only - http://bmjopen.bmj.com/site/about/guidelines.xhtml

- To evaluate the effect of a 0+1 PCV schedule on NP carriage during the first two years of life, comparing a) PCV10-vaccinated and b) PCV13-vaccinated participants with unvaccinated controls
- To evaluate the effect of a 1+1 PCV schedule on NP carriage during the first two years of life, comparing a) PCV10-vaccinated and b) PCV13-vaccinated participants with unvaccinated controls

Secondary objectives

- To evaluate the immunogenicity of a 0+1 schedule of PCV10 or PCV13
- To evaluate the immunogenicity of a 1+1 schedule of PCV10 or PCV13
- To determine whether the response to a dose of PCV10 or PCV13 at 12 months of age is enhanced by a dose at 2 months of age (i.e. 0+1 vs. 1+1)
- To investigate the kinetics of the immune response to PCV vaccination at 12 months of age, comparing antibody levels and memory B cell responses 7- and 28-days post-vaccination
- To examine the serotype profile of transferred maternal pneumococcal antibodies at 2 months of age
- To directly compare PCV10 and PCV13 in terms of immune responses and effect on NP carriage

Trial design

VPT-II is a single-blind, open-label, randomised controlled phase II/III clinical trial to investigate simplified PCV schedules that are focussed primarily on the generation of herd immunity. Participants are randomised to one of five groups: Group V receives a 0+1 PCV10 schedule, Group W receives a 0+1 PCV13 schedule, Group X receives a 1+1 PCV10 schedule, Group Y receives a 1+1 PCV13 schedule, and Group Z is a control group (that receives a single dose of PCV10 at 24 months of age). This trial is registered with ClinicalTrials.gov (NCT03098628), and the trial registration data set can be found in Appendix 1.

METHODS

Study setting

For peer review only - http://bmjopen.bmj.com/site/about/guidelines.xhtml

The trial is conducted in three districts within Ho Chi Minh City, Vietnam (Districts 4, 7 and 8). Districts are divided into communes, each of which has a health centre that provides preventive health services including EPI vaccines, along with primary healthcare services. The trial is conducted in one commune health centre in each district, with participants drawn from the surrounding communes within that district.

Eligibility criteria

Inclusion criteria

In order to be eligible, subjects must meet all of the following criteria: aged between 2 months and 2 months plus 2 weeks, no significant maternal or perinatal history, born at or after 36 weeks gestation, written and signed informed consent from parent/legal guardian, lives within approximately 30 minutes of the commune health centre, and family anticipates living in the study area for the next 22 months.

Exclusion criteria

Subjects meeting any of the following criteria will not be eligible to participate: known allergy to any component of the vaccine, allergic reaction or anaphylactic reaction to any previous vaccine, known immunodeficiency disorder, known HIV-infected mother, known thrombocytopenia or coagulation disorder, administration or planned administration of any immunoglobulin or blood product since birth, severe birth defect requiring ongoing medical care, chronic or progressive disease, seizure disorder, history of severe illness, receipt of any 2 month vaccines through the EPI program, or family plans on giving the infant *Quinvaxem* (diphtheria, tetanus, pertussis, *Haemophilus influenzae* type b, hepatitis B vaccine; DTP-Hib-HBV).

Interventions

The interventions are PCV10 and PCV13. There are four intervention groups, which receive: PCV10 at 12 months of age (0+1 PCV10, Group V), PCV13 at 12 months of age (0+1 PCV13, Group W), PCV10 at 2 and 12 months of age (1+1 PCV10, Group X), or PCV13 at 2 and 12 months of age (1+1 PCV13, Group Y). Control group participants (Group Z) receive a single dose of PCV10 at 24 months of age. PCV is administered by intramuscular injection into the anterolateral thigh in children less than 18 months old and in the deltoid muscle of the arm in children aged 18 months

For peer review only - http://bmjopen.bmj.com/site/about/guidelines.xhtml

and over. All vaccinations are performed by Ministry of Health nurses specifically trained in infant vaccine administration. Single-dose vials of PCV10 and single-dose pre-filled syringes of PCV13 are used.

Criteria for discontinuing or modifying allocated interventions

There is no modification of doses for participants in this study. If a participant has an allergic or anaphylactic response to vaccination, they will be withdrawn from the study. Participants may also be withdrawn voluntarily by the parent/legal guardian at any time, or by the study staff if they refuse any further study procedures or develop any of the exclusion criteria during the course of the study.

Strategies to improve and monitor adherence

Scheduled visit dates are noted on a health record card kept by the parent. If a participant does not attend a scheduled visit, a reminder phone call is made from the study clinic. If the participant cannot be contacted directly, their local Commune Health Centre is contacted for further follow up by phone or by home visit.

Relevant concomitant care

Participants also receive four doses of *Infanrix-hexa*, which is a popular choice of DTP-containing vaccine but is only available on the private market, instead of the routine EPI vaccine *Quinvaxem*. With the exception of *Quinvaxem*, other vaccines are permitted in this study providing there are two weeks between the administration of other vaccines and study vaccines. Other medications are also permitted, with the exception of immunosuppressive medication and medications listed as contraindicated to the study vaccines.

Outcomes

Primary outcome measure

The primary outcome measure to address each of the co-primary objectives is carriage of VT pneumococci, defined as carriage of serotypes 1, 4, 5, 6B, 7F, 9V, 14, 18C, 19F or 23F for the PCV10 groups, or carriage of serotypes 1, 3, 4, 5, 6A, 6B, 7F, 9V, 14, 18C, 19A, 19F or 23F for the PCV13 groups. The VT pneumococcal carriage prevalence (defined as the proportion of participants positive for VT pneumococcal carriage) will be determined at 24 months of age (primary endpoint), and also at 6, 12 and 18 months of age (secondary endpoints). Traditional culture methods (colonial morphology, α-haemolysis and the optochin test) and serotyping by latex agglutination/Quellung, with *lytA* PCR confirmation of nonencapsulated

For peer review only - http://bmjopen.bmj.com/site/about/guidelines.xhtml

isolates, will be the main methods used to analyse NP swabs collected at 6 and 12 months of age. Quantitative real-time PCR (qPCR) targeting the *lytA* gene[15] and serotyping by microarray[16] will be the main methods used to analyse NP swabs collected at 18 and 24 months of age.

Secondary microbiological outcome measures

- Carriage of any pneumococcal serotype at 6, 12, 18 and 24 months of age
- Non-VT pneumococcal carriage at 6, 12, 18 and 24 months of age
- Serotype-specific pneumococcal carriage at 6, 12, 18 and 24 months of age
- Density of pneumococcal carriage (overall, VT, non-VT and serotype-specific) at 18 and 24 months of age

Secondary immunological outcome measures

- Serotype-specific IgG antibody concentrations for all PCV13 serotypes, measured by ELISA[17] from all blood samples
- Opsonisation indices (OI) for all PCV13 serotypes, measured by opsonophagocytic assay (OPA)[18] for 50 participants per intervention group (Groups V-Y) pre- and four weeks post-12 months dose of PCV
- Polysaccharide specific memory B cells for serotypes 1, 5, 6A, 6B, 14, 19A and 23F, enumerated by ELISPOT[19] for 50 participants per intervention group (Groups V-Y) pre-, 7 days post- and 28 days post-12 months dose of PCV and at 24 months of age

An overview of the procedures for collection, transportation and laboratory analyses of the blood and NP samples can be found in Appendix 2.

Participant timeline

Participants are enrolled at 2 months of age and followed up to 24 months of age (Table 1a). Group V-Y participants provide NP swabs at 6 and 12 months of age, and all participants provide NP swabs at 18 and 24 months of age for analysis of the NP carriage outcomes. A subset of 200 participants per group will be included in the immunology sub-study and provide three (Groups V-Y) or one (Group Z) blood sample(s) over the course of the trial for analysis of vaccine responses (Table 1b).

Table 1a: Schedule of enrolment, interventions and assessments

For peer review only

Age (months)	2m	3m	4m	6m	12m	18m	24m
ENROLMENT:							
Informed consent	✓						
Eligibility assessment	✓						
Allocation	✓						
INTERVENTIONS:							
PCV10	X				V, X		Z
PCV13	Y				W, Y		
Infanrix-hexa	✓	✓	✓			✓	
ASSESSMENTS:							
Demographics	✓						
Household characteristics	✓						
Nasopharyngeal swab				V-Y	V-Y	✓	✓
General health	✓	✓	✓	✓	✓	✓	✓

✓ indicates applies to all groups (V-Z), otherwise group(s) specified.

Table 1b: Schedule of blood samples for immunology sub-study (subset of Groups V-Z)

Age (months)	2m	3m	12m	Post-12m[1]	24m
Sub-group a			V-Y[2]	V-Y	V-Y
Sample volume			7.5ml	2ml	3.5ml
Assays			ELISA, B cell	ELISA	ELISA
Sub-group b	V	W-Y	V-Y	V-Y	
Sample volume	2ml	2ml	2ml	7.5ml	
Assays	ELISA	ELISA	ELISA	ELISA, B cell	
Sub-group c			V-Y	V-Y	V-Y
Sample volume			3.5ml	7.5ml	3.5ml
Assays			ELISA	ELISA, B cell	ELISA
Sub-group d			V-Y	V-Y	V-Y
Sample volume			3.5ml	3.5ml	7.5ml
Assays			ELISA, OPA	ELISA, OPA	ELISA, B cell
Sub-group Z-I					Z
Sample volume					3.5ml
Assays					ELISA

[1] The post-12m blood sample is collected at 12 months plus 7 days in sub-groups a and b and at 12 months plus 28 days in sub-groups c and d. [2] 3.5ml sample for ELISA only in group W.

Sample size

The target sample size is 2500 with an allocation ratio of 4:4:4:4:9. This allocation ratio provides the greatest power for a total sample size of 2500 and results in target group sizes of 400 for each of the four different infant vaccination schedules (Groups V-Y) and 900 for the control group (Group Z). Sample size calculations are based on the primary outcome of effect on VT pneumococcal carriage at 24 months of age. For each of the four vaccination schedules compared with the unvaccinated control

For peer review only - http://bmjopen.bmj.com/site/about/guidelines.xhtml

group, the proposed sample size provides 82% power to detect a 40% reduction in the vaccinated group, with a two-sided type I error rate of 5%. The sample size calculations assume 15% VT pneumococcal carriage among controls, based on data from our previous pneumococcal vaccine trial in Ho Chi Minh City,[20] and 10% loss-to-follow-up.

Recruitment

Participants are recruited from infants born in the study communes during the enrolment period. Potential participants are identified from commune health centre birth records and are visited by commune health centre staff when they are approximately six weeks old. Verbal and written information about the trial is provided in Vietnamese and those interested in participating are referred to the study clinic when the infant is approximately two months old. At this time, written informed consent is obtained (Appendix 3), after which a study nurse/doctor examines the infant to ensure that all the eligibility criteria are met. Recruitment rates will be monitored on a monthly basis and meetings held with study staff and commune health centre staff to discuss any significant declines in recruitment rates.

Allocation

The allocation ratio of Groups V, W, X, Y and Z is 4:4:4:4:9. The first 200 participants enrolled into each of Groups V-Y, and a randomly selected 200 of the first 450 participants concurrently enrolled into Group Z, will go into the immunology sub-study. These participants (with the exception of Group Z) will be further randomised into one of four sub-groups (a, b, c or d) in an equal allocation ratio (Table 1b). Randomisation will be conducted by a database manager in Australia, using a computer-generated list of random numbers in a block randomisation scheme stratified by district. The group allocation, and sub-group allocation where relevant, is contained within a sealed envelope at the study clinic. Participants are assigned to a study group by a study doctor, using the next available envelope. The envelopes are prepared and sealed in the study office in Vietnam, by staff with no involvement in the recruitment process.

Blinding

All outcome measures are laboratory-based. Laboratory staff are blinded to the study group allocation. Laboratory samples are labelled with a unique ID number, which does not identify the study group. Given the different timing of the vaccination

For peer review only - http://bmjopen.bmj.com/site/about/guidelines.xhtml

schedules in the different groups, the study nurses, vaccine administrators and participants will not be blinded to the study group allocation.

Data collection methods

Data collected at the clinic are documented by dedicated, trained study staff using standardised forms. Blood samples and NP swabs are collected by staff specifically trained in the collection of samples from infants, and the volume of blood and swab quality are recorded. Laboratory data generated in both Vietnam and Australia are entered directly into dedicated databases in the laboratories by the laboratory staff and sent periodically to the data management team in Australia.

Retention

Appointments are documented on a parent-held health record card and a reminder phone call made the week before the scheduled visit. Missed visits are re-booked by phone and participants who miss a study visit will continue to be followed up for both sample collection and vaccine administration where possible. Participants receive a small payment at each visit towards transport costs.

Data management

Data collected at the clinic are entered on-site into a secure, web-based electronic data capture system, REDCap.[21] All entered data are monitored against the source documents for accuracy and completeness, and a series of data checks are performed on a regular basis to identify potential errors in the data. An audit trail of corrections and changes to the data is stored within the REDCap database. Immunology results are double-entered in a Microsoft Excel spreadsheet. NP culture results are entered in a Microsoft Access database and qPCR and microarray results are exported from SentiNET into Microsoft Excel or REDCap databases. The data collection forms and laboratory results are linked at the time of analysis.

Statistical methods

Analysis of primary and secondary outcomes

The primary outcome of VT carriage will be presented as a proportion (prevalence). VT carriage prevalence in each of the vaccinated groups (PCV10-type carriage for Groups V and X and PCV13-type carriage for Groups W and Y) will be compared with unvaccinated controls. Prevalence ratios (ratio of prevalence in each vaccinated

For peer review only - http://bmjopen.bmj.com/site/about/guidelines.xhtml

group to prevalence in unvaccinated controls) with 95% confidence intervals will be calculated, and groups compared using chi-squared tests (two-sided p-values reported). The primary endpoint is 24 months of age, with secondary endpoints of 6, 12, and 18 months of age. At 6 and 12 months of age, time points at which no swabs were collected from Group Z, swabs from the 0+1 groups (Groups V and W combined) will form the unvaccinated comparator.

Immunological outcomes will be summarised in terms of: geometric mean concentrations (GMCs) and the proportion with protective antibody levels (≥0.35μg/mL)[22] with 95% CIs for serotype-specific IgG data, geometric mean opsonisation indices (GMOIs) and the proportion with an OI ≥8[23] with 95% CIs for OPA data, and the mean number of antibody secreting cells per 10^6 PBMCs for polysaccharide-specific memory B cell data. GMC and GMOI ratios and risk differences with 95% CIs will also be calculated. Means will be compared using T-tests and proportions compared using Fisher's Exact tests.

Populations of analysis

Analysis is planned on an intention-to-treat population (ITT), with all participants to be analysed in the group to which they were randomised. Withdrawn participants may not contribute data at all time points as blood and NP samples may not be collected after their withdrawal.

Data monitoring

Data monitoring committee

Safety oversight is under the direction of an independent Data Safety and Monitoring Board (DSMB), in accordance with a DSMB Charter kept in the trial office. The DSMB will consist of at least three members, including two physicians and one biostatistician. The DSMB will meet periodically to review aggregate and individual participant data related to safety, data integrity and overall conduct of the trial, including a detailed review of all Serious Adverse Events (SAEs).

Interim analyses and stopping guidelines

No interim analyses are planned. Statistical rules will not be used to halt study enrolment or vaccine administration. Stopping guidelines are based on safety. An extraordinary meeting of the DSMB will be called in the event that serious safety

For peer review only

For peer review only - http://bmjopen.bmj.com/site/about/guidelines.xhtml

issues emerge, to provide recommendations regarding termination of the trial. A final decision to terminate rests with the Principal Investigators and the Sponsor.

Harms

Data on SAEs will be collected throughout the duration of the main study, with parents asked about hospitalisations and significant signs and symptoms at each study visit and through a regular review of hospital records. All SAEs will be recorded on the standard Vietnam Ministry of Health reporting form and reported to the Principal Investigators and the Ethics Committees. Participants will be kept under observation for 30 minutes following vaccine administration to monitor for any adverse reactions, and adverse events that may contraindicate further vaccinations will be assessed following all vaccination visits. Reactogenicity will be assessed following all doses of PCV using parent held diary cards.

Auditing

External site monitoring will be provided by Family Health International (FHI360), to independently assess protocol and GCP compliance. The frequency of monitoring visits and the level of detail of the monitoring will be documented in a contract between FHI360 and the sponsor. Monitoring visits will start prior to enrolment of the first participant and continue through to study-close-out.

Patient and public involvement

Patients were not involved in the development, design, recruitment or conduct of the study. Community consultation took place at the district level during the design phase, as well as discussion and approval of the design from the district health centres, the city Department of Health, and the People's Committee of Ho Chi Minh City. Participants will be informed of the overall study results by email or by post, with addresses collected at the final study visit.

ETHICS AND DISSEMINATION

Research ethics approval

The protocol and the Informed Consent Form (ICF) have approval from the Institutional Review Board at the Pasteur Institute of Ho Chi Minh City, the Vietnam Ministry of Health Ethical Review Committee for Bio-medical Research and the Human Research Ethics Committee of the Royal Children’s Hospital, Melbourne.

For peer review only

Both Ethics Committees receive annual reports on the trial progress, for continuing approval of the trial.

Protocol amendments

Any modifications to the protocol that may impact on the conduct of the study will be documented in a formal protocol amendment and approved by both Ethics Committees prior to implementation of the changes. The modified protocol will be given a new version number and date. The Ethics Committees will also be notified of any minor corrections/clarifications or administrative changes to the protocol, which will be documented in a protocol amendment letter. Significant protocol changes will also be updated in the ClinicalTrials.gov record.

Consent

Obtaining consent

The consent process is undertaken by specifically trained study staff. The study staff go through the ICF, translated into Vietnamese, in detail with the potential participant's parent/legal guardian. Study staff then discuss the trial further and answer any questions that may arise. Written informed consent is required prior to enrolment of the infant into the study and is provided by the parent/legal guardian as the participants are too young to provide consent themselves. A copy of the ICF will be given to the parent/legal guardian for their records.

Ancillary studies

Specific consent for the indefinite storage of blood and NP samples for future research related to the trial will be obtained from the parent/legal guardian and recorded on the ICF. Any future research will undergo ethical review. Any samples for which indefinite storage is not consented to will be destroyed at the close of the study.

Confidentiality

All study-related information will be stored securely and held in strict confidence. All documents kept at the study clinics are stored in locked cabinets. The REDCap database is password protected. All documents maintained centrally are stored in the trial office, which is kept locked. The laboratory samples and electronic laboratory data are coded by participant ID number and do not contain names. Access to participants' information will be granted to FHI360 for monitoring purposes, and to the Ethics Committees or DSMB if required.

For peer review only - http://bmjopen.bmj.com/site/about/guidelines.xhtml

Declaration of Interests

KM, CS and CN are investigators on a collaborative study on PCV impact on adult pneumonia funded by Pfizer.

Access to data

The final dataset will be under the custody of the trial sponsor, MCRI. The Principal Investigator, trial manager and trial statistician will have access to the full anonymised final dataset.

Ancillary and post-trial care

Participants are advised to come to the study clinic for ancillary care, or to Children's Hospital Number 1 or Children's Hospital Number 2 in Ho Chi Minh City, where they will not be charged for treatment and services. All participants are covered by clinical trials insurance for trial related harms.

Dissemination policy

Plans

Following completion of the trial, the results will be submitted for publication in peer-reviewed journals and presented at relevant international conferences. The results will be disseminated regardless of the magnitude or direction of effect. This research is undertaken as a collaboration between MCRI and the Pasteur Institute of Ho Chi Minh City. Either party must obtain the prior approval of the other party in advance of submitting a manuscript for publication, and such approval will not be unreasonably withheld.

Authorship

A small group of senior investigators will consider all proposed publications, with the final decision on content and authorship resting with the Principal Investigator. The role of each author will be published in line with journal requirements. Group authors may be used where appropriate. There are no plans for the use of professional writers.

Reproducible research

Data will be made publicly available in accordance with the rules set out by the Bill & Melinda Gates Foundation.

For peer review only

AUTHORS' CONTRIBUTIONS

BT wrote the first draft of this manuscript and drafted the trial protocol with input from HPT, KB and DYU. HPT also advised on the study design and oversaw the approval processes in Vietnam. VTTD, AB, PVL, CS and HSV were involved in the laboratory-related aspects of the design and protocol development. CDN advised on the study design and statistical aspects of the trial. TVN was involved in the design and establishment of the trial and had overall responsibility for its conduct in Vietnam as the site Principal Investigator. KM conceived the study, provided oversight for all aspects of the design and implementation, and had overall responsibility for the trial as Principal Investigator. All authors contributed to refinement of the trial protocol and reviewed and approved this manuscript.

ACKNOWLEDGEMENTS AND FUNDING STATEMENT

We thank the study participants and their families and the study staff. We acknowledge the contributions of Phan Trong Lan, Sophie La Vincente, Helen Thomson and Thi Que Huong Vu (deceased). This work is supported by the Bill & Melinda Gates Foundation (grant number OPP-1116833/INV-008627). We also acknowledge the Victorian Government's Operational Infrastructure Support Program.

COMPETING INTERESTS STATEMENT

All authors receive salary support from grants from the Bill & Melinda Gates Foundation. KM, CS and CN are investigators on a collaborative study on PCV impact on adult pneumonia funded by Pfizer. None of the authors have any other competing interests to declare.

APPENDICES

Appendix 1 – Administrative information

Appendix 2 – Sample collection and processing

Appendix 3 - Informed Consent Form. These materials were translated into Vietnamese.

For peer review only

References

1. Collaborators GBDLRI. Estimates of the global, regional, and national morbidity, mortality, and aetiologies of lower respiratory infections in 195 countries, 1990-2016: a systematic analysis for the Global Burden of Disease Study 2016. *The Lancet. Infectious diseases* 2018;18:1191-210. doi:10.1016/S1473-3099(18)30310-4.
2. Myint TT, Madhava H, Balmer P, et al. The impact of 7-valent pneumococcal conjugate vaccine on invasive pneumococcal disease: a literature review. *Advances in therapy* 2013;30:127-51. doi:10.1007/s12325-013-0007-6.
3. Conklin L, Loo JD, Kirk J, et al. Systematic review of the effect of pneumococcal conjugate vaccine dosing schedules on vaccine-type invasive pneumococcal disease among young children. *Pediatr Infect Dis J* 2014;33 Suppl 2:S109-18. doi:10.1097/INF.0000000000000078.
4. Loo JD, Conklin L, Fleming-Dutra KE, et al. Systematic review of the effect of pneumococcal conjugate vaccine dosing schedules on prevention of pneumonia. *Pediatr Infect Dis J* 2014;33 Suppl 2:S140-51. doi:10.1097/INF.0000000000000082.
5. Shiri T, Datta S, Madan J, et al. Indirect effects of childhood pneumococcal conjugate vaccination on invasive pneumococcal disease: a systematic review and meta-analysis. *The Lancet. Global health* 2017;5:e51-e59. doi:10.1016/S2214-109X(16)30306-0.
6. Pneumococcal conjugate vaccines in infants and children under 5 years of age: WHO position paper - February 2019. *Wkly Epidemiol Rec* 2019;94:85-104.
7. Goldblatt D, Southern J, Andrews NJ, et al. Pneumococcal conjugate vaccine 13 delivered as one primary and one booster dose (1 + 1) compared with two primary doses and a booster (2 + 1) in UK infants: a multicentre, parallel group randomised controlled trial. *The Lancet. Infectious diseases* 2017. doi:10.1016/S1473-3099(17)30654-0.
8. Whitney CG, Pilishvili T, Farley MM, et al. Effectiveness of seven-valent pneumococcal conjugate vaccine against invasive pneumococcal disease: a matched case-control study. *Lancet* 2006;368:1495-502. doi:10.1016/S0140-6736(06)69637-2.
9. Andrews NJ, Waight PA, Burbidge P, et al. Serotype-specific effectiveness and correlates of protection for the 13-valent pneumococcal conjugate vaccine: a postlicensure indirect cohort study. *The Lancet. Infectious diseases* 2014;14:839-46. doi:10.1016/S1473-3099(14)70822-9.
10. Huebner RE, Mbelle N, Forrest B, et al. Immunogenicity after one, two or three doses and impact on the antibody response to coadministered antigens of a nonavalent pneumococcal conjugate vaccine in infants of Soweto, South Africa. *Pediatr Infect Dis J* 2002;21:1004-7. doi:10.1097/01.inf.0000036012.37665.fe.
11. Russell FM, Balloch A, Tang ML, et al. Immunogenicity following one, two, or three doses of the 7-valent pneumococcal conjugate vaccine. *Vaccine* 2009;27:5685-91. doi:10.1016/j.vaccine.2009.06.098.
12. Russell FM, Carapetis JR, Burton RL, et al. Opsonophagocytic activity following a reduced dose 7-valent pneumococcal conjugate vaccine infant primary series and 23-valent pneumococcal polysaccharide vaccine at 12 months of age. *Vaccine* 2011;29:535-44. doi:10.1016/j.vaccine.2010.10.046.
13. Licciardi PV, Temple B, Dai VTT, et al. Immunogenicity of alternative ten-valent pneumococcal conjugate vaccine schedules in infants in Ho Chi Minh City, Vietnam: results from a single-blind, parallel-group, open-label, randomised, controlled trial. *The Lancet. Infectious diseases* 2021. doi:10.1016/S1473-3099(20)30775-1.

For peer review only - http://bmjopen.bmj.com/site/about/guidelines.xhtml

14. Russell FM, Licciardi PV, Balloch A, et al. Safety and immunogenicity of the 23-valent pneumococcal polysaccharide vaccine at 12 months of age, following one, two, or three doses of the 7-valent pneumococcal conjugate vaccine in infancy. *Vaccine* 2010;28:3086-94. doi:10.1016/j.vaccine.2010.02.065.
15. Satzke C, Dunne EM, Choummanivong M, et al. Pneumococcal carriage in vaccine-eligible children and unvaccinated infants in Lao PDR two years following the introduction of the 13-valent pneumococcal conjugate vaccine. *Vaccine* 2019;37:296-305. doi:10.1016/j.vaccine.2018.10.077.
16. Dunne EM, Satzke C, Ratu FT, et al. Effect of ten-valent pneumococcal conjugate vaccine introduction on pneumococcal carriage in Fiji: results from four annual cross-sectional carriage surveys. *The Lancet. Global health* 2018;6:e1375-e85. doi:10.1016/S2214-109X(18)30383-8.
17. Balloch A, Licciardi PV, Leach A, et al. Results from an inter-laboratory comparison of pneumococcal serotype-specific IgG measurement and critical parameters that affect assay performance. *Vaccine* 2010;28:1333-40. doi:10.1016/j.vaccine.2009.11.011.
18. Balloch A, Roalfe L, Ekstrom N, et al. Interlaboratory Comparison of the Pneumococcal Multiplex Opsonophagocytic Assays and Their Level of Agreement for Determination of Antibody Function in Pediatric Sera. *mSphere* 2018;3. doi:10.1128/mSphere.00070-18.
19. Licciardi PV, Toh ZQ, Clutterbuck EA, et al. No long-term evidence of hyporesponsiveness after use of pneumococcal conjugate vaccine in children previously immunized with pneumococcal polysaccharide vaccine. *The Journal of allergy and clinical immunology* 2016;137:1772-79 e11. doi:10.1016/j.jaci.2015.12.1303.
20. Temple B, Nation ML, Dai VTT, et al. Effect of a 2+1 schedule of ten-valent versus 13-valent pneumococcal conjugate vaccine on pneumococcal carriage: Results from a randomised controlled trial in Vietnam. *Vaccine* 2021;39:2303-10. doi:10.1016/j.vaccine.2021.02.043.
21. Harris PA, Taylor R, Thielke R, et al. Research electronic data capture (REDCap)--a metadata-driven methodology and workflow process for providing translational research informatics support. *Journal of biomedical informatics* 2009;42:377-81. doi:10.1016/j.jbi.2008.08.010.
22. WHO Expert Committee on Biological Standardization. WHO Technical Report Series 927. Geneva: World Health Organisation, 2005.
23. Burton RL, Nahm MH. Development and validation of a fourfold multiplexed opsonization assay (MOPA4) for pneumococcal antibodies. *Clin Vaccine Immunol* 2006;13:1004-9. doi:13/9/1004 [pii]
10.1128/CVI.00112-06.

For peer review only

APPENDICES

Table of contents

APPENDIX 1: Administrative information ... 2

APPENDIX 2: Sample collection and processing ... 5

1 SAMPLE COLLECTION ... 5

2 LABORATORY EVALUATIONS OF NASOPHARYNGEAL SWABS ... 6

2.1 TRADITIONAL CULTURE METHODS ... 6
2.2 QPCR AND MICROARRAY ... 7

3 LABORATORY EVALUATIONS OF BLOOD SAMPLES ... 7

3.1 ELISAS ... 7
3.2 OPAS ... 8
3.3 B CELL ASSAYS ... 8

4 REFERENCES ... 9

APPENDIX 3: Information statement and Consent form ... 10

PART I: INFORMATION STATEMENT ... 10

PART II: CONSENT FORM ... 14

For peer review only

APPENDIX 1
Administrative information

Title
A randomised controlled trial of simplified 0+1 and 1+1 pneumococcal vaccine schedules in Ho Chi Minh City, Vietnam

Trial registration
ClinicalTrials.gov: NCT03098628

Trial registration - data set

Data category	Information
Primary registry and trial identifying number	ClinicalTrials.gov NCT03098628
Date of registration in primary registry	1 March 2017
Secondary identifying numbers	HREC36027
Source(s) of monetary or material support	Bill & Melinda Gates Foundation
Primary sponsor	Murdoch Children's Research Institute, Australia
Contact for public queries	Professor Kim Mulholland kim.mulholland@lshtm.ac.uk
Contact for scientific queries	Professor Kim Mulholland kim.mulholland@lshtm.ac.uk
Public title	A randomised controlled trial of simplified 0+1 and 1+1 pneumococcal vaccine schedules in Ho Chi Minh City, Vietnam
Scientific title	Trial of simplified pneumococcal vaccination in Vietnam II (VPT-II): the herd immunity approach
Countries of recruitment	Vietnam
Health condition(s) or problem(s) studied	Pneumococcal vaccination responses
Intervention(s)	Active Comparator V: PCV10 administered at 12 months of age (0+1 PCV10)
	Active Comparator W: PCV13 administered at 12 months of age (0+1 PCV13)
	Active Comparator X: PCV10 administered at 2 and 12 months of age (1+1 PCV10)
	Active Comparator Y: PCV13 administered at 2 and 12 months of age (1+1 PCV13)
	Control Z: PCV10 administered at end of trial (24 months)

For peer review only

Key inclusion and exclusion criteria	Inclusion criteria:<br>• aged between 2 months and 2 months plus 2 weeks<br>• no significant clinical maternal or perinatal history<br>• born at or after 36 weeks' gestation<br>• written and signed informed consent from parent/legal guardian<br>• lives within approximately 30 minutes of the commune health centre<br>• family anticipates living in the study area for the next 22 months
	Exclusion criteria:<br>• known allergy to any component of the vaccine<br>• allergic reaction or anaphylactic reaction to any previous vaccine<br>• known immunodeficiency disorder<br>• known HIV-infected mother<br>• known thrombocytopenia or coagulation disorder<br>• administration or planned administration of any immunoglobulin or blood product since birth<br>• severe birth defect requiring ongoing medical care<br>• chronic or progressive disease; seizure disorder<br>• history of severe illness<br>• receipt of any 2 month vaccines through the EPI program<br>• family plans on giving the infant *Quinvaxem* (DTP-Hib-HBV)
Study type	Interventional, randomised, parallel group, open label phase II/III trial. Outcome assessors (laboratory) blinded. Purpose: prevention.
Enrolment period	8 March 2017 – 11 June 2020
Sample size	Target: 2500<br>Number enrolled: 2501
Recruitment status	Active, not recruiting
Primary outcome	Vaccine-type (VT) pneumococcal carriage at 24 months of age
Key secondary outcomes	• VT pneumococcal carriage at 6, 12 and 18 months of age<br>• Non-VT pneumococcal carriage at 6, 12, 18 and 24 months of age<br>• Carriage of any pneumococcal serotype at 6, 12, 18 and 24 months of age<br>• Serotype-specific IgG antibody concentrations post-2-month dose, pre-12-month dose, post-12-month dose of PCV and at 24 months of age<br>• Serotype-specific opsonophagocytic indices pre- and post-12-month dose of PCV<br>• Serotype-specific memory B cell numbers pre- and post-12-month dose of PCV and at 24 months of age
Ethics Review	Approved by the Human Research Ethics Committee of the Royal Children's Hospital Melbourne and the Vietnam Ministry of Health Ethics Committee

For peer review only

Protocol version

Protocol version 5.0 dated 8 February 2018

Revision chronology
Original: Version 3.2, 11 October 2016.
First amendment: Version 4.0, 16 May 2017. Main reason for amendment: minor clarifications requested by the Vietnam Ministry of Health, along with a change to a final version number
Second amendment: Version 5.0, 8 February 2018. Main reason for amendment: to remove references to Japanese Encephalitis Vaccine (JEV) and measles-rubella (MR) vaccine, as for logistical reasons these are to be administered through the Commune Health Centres and not as part of the study

Roles and responsibilities

Sponsor contact information
Trial Sponsor: Murdoch Children's Research Institute, Royal Children's Hospital, Flemington Road, Parkville, Victoria 3052, Australia
Telephone: +61 3 8341 6200
Contact name: Professor Kim Mulholland

Other Institutions
Menzies School of Health Research, Darwin, Australia
Role: oversight of traditional microbiology (culture methods)

Family Health International (FHI360), Ho Chi Minh City, Vietnam
Role: external clinical trial monitoring

Bill & Melinda Gates Foundation
Role: funding source
The funder does not have any role in the trial conduct, trial management, laboratory tests, or data analyses.

For peer review only

APPENDIX 2
Sample collection and processing

1 Sample Collection

Participants will provide four (Groups V-Y) or two (Group Z) nasopharyngeal (NP) swabs over the course of the trial. Additionally, participants in the immunology sub-study will provide three (Groups V-Y) or one (Group Z) venous blood sample(s) over the course of the trial. A minimum of two members of the study team will be present during the collection of NP swabs and blood samples, to ensure no injury is caused by an infant's sudden movement. Distraction techniques will be utilised to minimise discomfort.

NP samples will be collected, stored and transported in line with World Health Organization (WHO) guidelines[1] using sterile nylon flocked swabs. Following collection, swab will be placed immediately into 1000 µL Skim Milk Tryptone Glucose Glycerol Broth (STGGB). The samples will be kept chilled until transportation to the Pasteur Institute, Ho Chi Minh City, Vietnam (Pasteur). On arrival at Pasteur two aliquots will be removed, and the aliquots and original sample will be frozen at ≤ -70°C, within 8 hours of collection. NP swabs collected at 18 and 24 months of age (NPs c and d) will be shipped to the Murdoch Children's Research Institute, Melbourne, Australia (MCRI) on dry ice and stored at ≤ -70°C prior to analysis.

Blood samples will be collected using a butterfly needle and vacutainer, or, if the infant's veins are difficult to palpate, using a syringe attached to a 23G Surflo winged infusion set or through a finger prick using an appropriate lancet. The volume of blood collected is: 2 mL at 2-3 months of age or for ELISA samples when two samples are collected 7 days apart; 7.5 mL for B cell assays; and 3.5 mL at other time points. 2 mL and 3.5 mL blood samples will be collected into gel vacutainer tubes and kept chilled until transportation to Pasteur in a transport cooler box, unless specific laboratory tests require alternative collection methods. On arrival at the laboratory the sera will be centrifuged in a fridge centrifuge then divided into up to four aliquots, stored in micro-tubes and frozen at ≤ -70°C prior to analysis. 7.5 mL blood samples will be collected into sodium heparin vacutainer tubes and transported to Pasteur at room temperature the same day. On arrival at Pasteur plasma and peripheral blood mononuclear cells (PBMCs) will be separated from each heparinized blood sample by density gradient centrifugation. Plasma will be divided into up to four aliquots and stored at ≤ -70°C prior to analysis. PBMCs will be counted and at least $10x10^6$ cells/mL will be used for B cell assays where indicated, and the remainder counted for viability using the trypan blue exclusion method. PBMCs will then be cryopreserved at ≤ -70°C in aliquots containing 8-$10x10^6$ cells/mL and stored until shipment to MCRI for further analysis.

For peer review only - http://bmjopen.bmj.com/site/about/guidelines.xhtml

2 Laboratory evaluations of nasopharyngeal swabs

Traditional microbiology will be used to analyse the NP swabs collected at 6 and 12 months of age (NPs a and b), and qPCR and microarray will be used to analyse the NP swabs collected at 18 and 24 months of age (NPs c and d). Traditional microbiology (the culturing of NP swabs and the identification and typing of *S. pneumoniae*) will be done at Pasteur, under guidance from the microbiology teams at Menzies School of Health Research, Darwin, Australia (Menzies) and MCRI. qPCR and microarray analysis will be performed at MCRI.

2.1 Traditional culture methods

Traditional culture methods were consistent with WHO guidelines.[1] Prior to analysis, batches of swabs will be removed from -70°C storage and thawed on ice. Once thawed, swabs will be vortexed for 10 seconds (sec) and 50 µL of each sample inoculated onto horse blood agar + colistin + nalidixic acid plates (horse blood CNA plates). Plates will be incubated overnight (18-24 hours) at 37°C in 5% CO_2. Identification of *S. pneumoniae* will primarily be based on colonial morphology (typically flat with a dimple 1-3 mm in size), α-haemolysis and the optochin test. One colony of the dominant morphology will be selected, along with an example of each morphologically distinct colony type. Colonies will be sub-cultured onto horse blood agar (HBA) to obtain pure isolates.

Serotyping of pneumococcal isolates will be performed by latex agglutination, with Quellung confirmation as required. Latex agglutination identifies pneumococcal serotypes by using sensitised latex particles.[2] The pneumococcal culture suspension is mixed with 10 µL of sensitised latex particles (produced in-house[3] from antisera obtained from the Statens Serum Institut) on clear glass slides and rotated for 2 minutes (min). A positive test is indicated by aggregation of latex particles and clearing of the suspension. Firstly, the isolate is screened with antisera pools and then with specific serogroup/type/factor reagents as required to determine the final serotype. Quellung serotyping will be used to resolve any inconclusive results from latex agglutination serotyping. Quellung uses antisera to determine the serogroup and serotype (including factor testing) of the pneumococcal isolate.[4] Pneumococcal culture (1 µL) is dotted onto a glass slide and mixed with 1 µL of antisera (Statens Serum Institute). Each drop is covered with a coverslip and examined by microscopy. A positive reaction is indicated by the appearance of capsular swelling. Any pneumococci that are non-typeable will be tested using PCR targeting the *lytA* gene to confirm species identification.[5]

For peer review only

2.2 qPCR and microarray

Each sample will be pelleted by centrifugation for 10 min at 6,000 x *g*. DNA will then be extracted from a 100 µL aliquot of STGGB, using a QIAcube HT (Qiagen) instrument and a QIAamp 96 DNA QIAcube HT Kit (Qiagen) with an initial pre-lysis step. The pre-lysis step includes a 30 min incubation at 37°C with a lysis buffer (20 mM Tris/HCl, 2 mM EDTA, 1% v/v Triton, 20 mg/mL lysozyme, 2 mg/mL RNase A, and 0.075 mg/mL mutanolysin), followed by a 30 min incubation at 56°C with 20 µL of Proteinase K and 200 µL of Buffer AL (Qiagen). qPCR targeting the *lytA* gene will be used to quantify pneumococcal density,[6] by reference to a standard curve from a dilution series of isolate genomic DNA (5 µL per well). Samples that are qPCR positive (cycle threshold (Ct) <35) or equivocal (CT 35-40) will be cultured on HBA containing 5 µg/mL gentamicin (gHBA, Oxoid). Samples that have α-haemolytic colonies will have growth harvested from the culture plates and DNA extracted on a QIAcube HT instrument (Qiagen) as described previously. Molecular serotyping will be conducted by microarray using the extracted DNA and Senti-SPv1.5 microarrays (BUGS Bioscience), with analysis using a custom web-based software.[7] Serotype-specific density will be calculated by multiplication of qPCR data (overall pneumococcal density) and microarray data (relative abundance of serotype(s)).

3 Laboratory evaluations of blood samples

All blood samples will be analysed by ELISA to measure serotype-specific anti-pneumococcal IgG antibody concentrations. A subset of blood samples from 12m and 12m+28d will also be analysed by OPA to measure functional serotype-specific anti-pneumococcal IgG, and a subset of blood samples from 12m, 12m+7d, 12m+28d and 24m will be analysed by ELISPOT assay to determine the memory B cell responses. ELISAs will be performed at Pasteur, under guidance from the immunology team at MCRI; OPAs will be performed at MCRI; and B cell assays will be performed at the Pasteur laboratory, with final reading of the plates performed at MCRI.

3.1 ELISAs

Serotype-specific anti-pneumococcal IgG will be measured for the serotypes in PCV13 using a previously published modified WHO ELISA method[8] and the new international reference serum, 007sp (FDA/CBER).[9] In brief, microtitre wells are coated with pneumococcal polysaccharide diluted in phosphate buffered saline (PBS). To neutralise non-specific antibodies, the reference serum 007sp, three controls and infant serum samples will be absorbed by overnight incubation in diluent containing cell wall polysaccharide and serotype 22F. Samples, controls and standard are loaded to the pre-coated plates and the assay is developed using HRP conjugated anti-human IgG. Detection is completed using a TMB (3,3_,5,5_-tetramethylbenzidine) substrate solution and the reaction stopped with 1M phosphoric acid. A high, medium, and low control serum will be included on each plate to assess assay performance and inter-assay variation. Results will be reported in µg/ml of serotype-specific IgG.

For peer review only

3.2 OPAs

OPA provides a measure of the opsonophagocytic and killing activity of anti- pneumococcal antibodies. Functional serotype-specific IgG will be measured for all serotypes in PCV13 using a multiplexed opsonophagocytic assay (MOPA),[10] a modification of the standardised single OPA.[11] Serial dilutions of heat inactivated infant sera are incubated with cultured HL-60 phagocytic cells (ATCC), rabbit Complement (Pel-Freez) and a mix of cultured antibiotic resistant *Streptococcus pneumoniae*. After 45 min, the serial dilutions are plated to selective Todd-Hewitt broth with Yeast Extract (THYE) agar plates. At 24 hours, the number of colonies per dilution is measured using a ProtoCol 3 colony counter. A control serum sample, a Complement control (no serum) and a bacterial control (no Complement) are included in each assay. Results are recorded as an opsonic index (OI), which is the reciprocal of the serum dilution with at least 50% killing when compared to the average growth in complement control wells. An OI ≥8 is considered a positive response. Negative results are recorded as OI=4.

3.3 B cell assays

The number of circulating memory B cells to pneumococcal serotypes will be determined by ELISPOT assay using a previously published method.[12] In brief, PBMCs are re-suspended in RPMI Foetal Calf Serum (FCS) at a concentration of $2x10^6$ cells/mL and 100µL added to each well of the culture plate containing an antigen cocktail (Staphylococcus aureus Cowan strain – Pansorbin cells (SAC; 1:5000), 2.5µg/mL CpG and 83ng/mL pokeweed mitogen). Plates are incubated at 37°C with 5% CO_2 and 95% humidity for 5 days. At day 5, cells are harvested and washed and the cell pellet re-suspended in 1mL RPMI-FCS and counted by trypan blue. Cells are then made up to a final concentration of $2x10^6$ cells/mL, seeded onto ELISPOT plates coated with anti-IgG (10µg/mL), tetanus toxoid (5µg/mL), diphtheria toxoid (10µg/mL) or pneumococcal polysaccharides conjugated to methylated human serum albumin (10-20µg/mL) and incubated overnight. Bound IgG is detected with an alkaline phosphatase-conjugated IgG and cells are counted using an automated ELISPOT reader and software. The total number of IgG-secreting antibody-forming cells (AFCs) is used as the positive control and 1,000 IgG AFCs/10^6 cultured PBMCs is the lower cut-off for inclusion in the analysis.

For peer review only

4 References

1. Satzke C, Turner P, Virolainen-Julkunen A, et al. Standard method for detecting upper respiratory carriage of *Streptococcus pneumoniae*: updated recommendations from the World Health Organization Pneumococcal Carriage Working Group. *Vaccine* 2013;32:165-79. doi:10.1016/j.vaccine.2013.08.062.
2. Porter BD, Ortika BD, Satzke C. Capsular Serotyping of *Streptococcus pneumoniae* by latex agglutination. *Journal of visualized experiments : JoVE* 2014:51747. doi:10.3791/51747.
3. Ortika BD, Habib M, Dunne EM, et al. Production of latex agglutination reagents for pneumococcal serotyping. *BMC Res Notes* 2013;6:49. doi:10.1186/1756-0500-6-49.
4. Habib M, Porter BD, Satzke C. Capsular serotyping of *Streptococcus pneumoniae* using the Quellung reaction. *Journal of visualized experiments : JoVE* 2014:e51208. doi:10.3791/51208.
5. World Health Organization. Laboratory methods for the diagnosis of meningitis caused by *Neisseria meningitidis, Streptococcus pneumoniae, and Haemophilus influenzae*: WHO manual. 2nd ed, 2011. WHO/IVB.11.09. Available at: https://apps.who.int/iris/handle/10665/70765
6. Satzke C, Dunne EM, Choummanivong M, et al. Pneumococcal carriage in vaccine-eligible children and unvaccinated infants in Lao PDR two years following the introduction of the 13-valent pneumococcal conjugate vaccine. *Vaccine* 2019;37:296-305. doi:10.1016/j.vaccine.2018.10.077.
7. Newton R, Hinds J, Wernisch L. Empirical Bayesian models for analysing molecular serotyping microarrays. *BMC Bioinformatics* 2011;12:88. doi:10.1186/1471-2105-12-88.
8. Balloch A, Licciardi PV, Leach A, et al. Results from an inter-laboratory comparison of pneumococcal serotype-specific IgG measurement and critical parameters that affect assay performance. *Vaccine* 2010;28:1333-40. doi:10.1016/j.vaccine.2009.11.011.
9. Goldblatt D, Plikaytis BD, Akkoyunlu M, et al. Establishment of a new human pneumococcal standard reference serum, 007sp. *Clin Vaccine Immunol* 2011;18:1728-36. doi:10.1128/CVI.05252-11.
10. Burton RL, Nahm MH. Development of a fourfold multiplexed opsonophagocytosis assay for pneumococcal antibodies against additional serotypes and discovery of serological subtypes in *Streptococcus pneumoniae* serotype 20. *Clin Vaccine Immunol* 2012;19:835-41. doi:10.1128/CVI.00086-12.
11. Rose CE, Romero-Steiner S, Burton RL, et al. Multilaboratory comparison of *Streptococcus pneumoniae* opsonophagocytic killing assays and their level of agreement for the determination of functional antibody activity in human reference sera. *Clin Vaccine Immunol* 2011;18:135-42. doi:10.1128/CVI.00370-10.
12. Licciardi PV, Toh ZQ, Clutterbuck EA, et al. No long-term evidence of hyporesponsiveness after use of pneumococcal conjugate vaccine in children previously immunized with pneumococcal polysaccharide vaccine. *The Journal of allergy and clinical immunology* 2016;137:1772-79 e11. doi:10.1016/j.jaci.2015.12.1303.

For peer review only

APPENDIX 3

Murdoch Childrens Research Institute

Information statement and consent form

PART I: INFORMATION STATEMENT

Research Project Title: **Vietnam Pneumococcal Trial II**

Principal Researcher:

Edward Kim Mulholland, MD
Nguyen Vu Thuong, MD

Research Partners:

Murdoch Childrens Research Institute, Melbourne, Australia
Pasteur Institute of Ho Chi Minh City, Ho Chi Minh City, Vietnam

HREC Project Number: 36027

Thank you for taking the time to read this **Parent/Guardian Information Statement and Consent Form**. We would like to invite your child to participate in a research project that is explained below.

This document is 5 pages long. Please make sure you have all the pages.

What is an Information Statement?

These pages tell you about the research project. It explains to you clearly and openly all the steps and procedures of the project. The information is to help you decide whether or not you would like your child to take part in the research. Please read this Information Statement carefully.

Before you decide if you want your child to take part or not, you can ask us any questions you have about the project. You may want to talk about the project with your family, friends or health care worker.

Important things you need to know

- It is your choice whether or not your child can take part in the research. You do not have to agree if you do not want to
- If you decide you do not want your child to take part, it will not affect the treatment and care your child receives at the CHC clinic.

If you would like your child to take part in the research project, please sign the consent form at the end of this information statement. By signing the consent form you are telling us that you:

- understand what you have read
- had a chance to ask questions and received satisfactory answers
- consent to your child taking part in the project

We will give you a copy of this information and consent form to keep.

For peer review only - http://bmjopen.bmj.com/site/about/guidelines.xhtml

Why are we doing the study?

Pneumonia is a common problem in Vietnam and throughout the developing world. In the developing world it is the leading cause of death in children under 5 years of age. A number of germs cause pneumonia but the most common germ is a bacteria called pneumococcus. Pneumococcus can also cause ear infections as well as other more severe diseases like meningitis (infection around the brain). This germ normally lives in the nose of humans and is spread from person to person by touching or sneezing. There are more than 90 types of this germ but only some types cause serious infections in young children.

Pneumococcal vaccines protect against infection with pneumococcus. There are two licensed pneumococcal vaccines. These are used in the United States and many countries in Europe. Unfortunately the costs of these vaccines are very high, so not all countries in the world can afford them. We are doing this study to find the best ways to protect babies from this germ and also to make it cheaper for countries like Vietnam to afford to buy the vaccine.

We hope that up to 2500 babies will take part in this study.

For peer review only

What does the study involve?

Consent: We will explain what is involved in the study and ask some questions about your baby's health. If you agree to join the study we will ask you to sign a consent form. After this, a study doctor will perform a health check of your baby to make sure your baby is healthy to take part.

Enrolment: Study doctors will examine all children to ensure that participants have no pre-existing health conditions that make them not eligible to take part in the study. To be enrolled in the study a child must:

- Be aged between 2 months and 2 months 2 weeks;
- Have been born at a gestation greater than 36 weeks in an uncomplicated pregnancy;
- Live within approximately 30 minutes from the study clinic and anticipate residing locally for the next 22 months;
- Parent / legal guardian has signed a consent form to participate in the research

Length of study: If you and your baby take part, you will need to come to this commune health centre for between seven and ten visits over 22 months. We will remind you when you need to come.

Questionnaire: At the start of the study you will be asked some questions about your family and your baby's health. These are to help us understand how the vaccines work best.

Health checks: Your baby will have a health check at each study visit. Your baby will have a more detailed health check at the 6 month and 12 month visits, involving physical and developmental assessments.

Vaccinations: There are five different vaccine groups in this study. Like rolling a dice your baby will be allocated to one of the five groups. Your baby will get one or two doses of pneumococcal vaccine, at the ages shown in the table below.

**Group** (number of participants)	**2 months**	**12 months**	**24 months**
**V** (400)		PCV10	
**W** (400)		PCV13	
**X** (400)	PCV10	PCV10	
**Y** (400)	PCV13	PCV13	
**Z** (900)			PCV10

Groups V and W receive a single dose at 12 months; X and Y receive 2 doses at 2 and 12 months and group Z does not receive PCV until 24 months of age.

PCV13 (*Prevnar-13*) covers 13 types of the pneumococcal germ and PCV10 (*Synflorix*) covers ten types of the pneumococcal germ.

For peer review only - http://bmjopen.bmj.com/site/about/guidelines.xhtml

Your baby will also get four doses of *Infanrix-hexa* 6-1, an infant vaccine that covers all the diseases that are covered by the standard vaccines used in Vietnam (diphtheria, tetanus, pertussis, hepatitis B, polio virus and *Haemophilus influenzae* type B). Note: Participants will receive the measles vaccine, two doses of Japanese Encephalitis vaccine and a dose of measles-rubella vaccine at their local Commune Health Centre, as per the routine EPI practice.

Nose swabs: Up to four nose swabs will be taken during the study, at 6, 12, 18 and 24 months of age. The nose swabs are to see if the vaccine will help stop the spread of the pneumococcus from child to child. This will involve putting a cotton wool swab (like a cotton bud) into your baby's nose for a couple of seconds. This may make your baby sneeze and possibly cry briefly – it tickles quite a lot, but doesn't really hurt.

Blood tests: Up to three blood tests will be taken during the study, at 12 months, 12 months plus 1 or 4 weeks, and at 2-3 months or at 24 months of age. The volume of blood taken will be 2ml at 2 months of age, 3.5ml at 3 months of age and 3.5ml or 7.5ml at other ages. The blood tests are to check the response to the vaccines. Blood will be taken by staff from Children's Hospital Number 1 or 2. If you like we can put local anaesthetic cream on your baby's skin before taking the blood test so that it doesn't hurt as much.

Hospitalisation: If your baby becomes unwell during the study, we may need to look at your child's medical records. If your baby is admitted to hospital with respiratory symptoms, a chest x-ray and/or a nose swab may be taken. If your baby is admitted to hospital with diarrhoea, a stool sample may be collected.

Benefits of the study

Pneumococcal vaccines are not presently available in the EPI program in Vietnam. By joining the study your baby will have some protection from diseases such as ear infections and pneumonia caused by the commonest pneumococcal germs. In addition children will receive 4 doses of *Infanrix-Hexa*

Are there any risks?

The vaccines we are using are safe and are licensed in many countries (PCV10 is licensed in Vietnam); therefore there is little danger to any child participating in the study. As with all vaccines, your baby may feel some pain or discomfort where the injection is given, and there is a small risk of soreness and redness. Some babies in the study will get one more injection than they would routinely get at 2 months of age. Children will be kept at the study clinic for 30 minutes after each injection to monitor for any unexpected reactions and provide treatment if required. Your baby may feel some pain or discomfort when the blood tests are taken, and there is small risk of bruising, swelling or minor bleeding.

Confidentiality

All information collected in this study will remain confidential and will be used for research purposes only. Your baby will be given an identification number at the start of the study. Any information collected will use this number and will not include your baby's name. All information will be kept secure, stored either in Vietnam or Australia. Information will be stored for at least 15 years after the study finishes.

Some of the samples we collect will be sent to overseas laboratories for tests. These laboratories will not be given your child's name. If you give permission we will keep your baby's blood and nose swab samples indefinitely for other similar tests in the future, either in Vietnam or Australia. This will help us to perform any new pneumococcal test that may be developed in the future.

The results of the study will be published in scientific journals and presented at conferences. There will never be details published that would identify your baby.

Monitors reporting to the donors and state authorities will have access to the research records of your child.

For peer review only - http://bmjopen.bmj.com/site/about/guidelines.xhtml

Withdrawal from the Study

You are free to withdraw your baby from the study at any time. This will not affect any of your baby's future health care treatment and there will be no harmful consequences for your baby. If your baby has not had all their pneumococcal vaccines they may not be fully protected against the pneumococcal germs which most commonly affect babies. However, they will still get some protection from any doses of vaccine received.

Compensation

We will pay 200,000VND towards the transport cost for coming to the clinic for each study visit.

What happens if my child is injured or becomes ill during the project?

If your child suffers any injury or complication as a result of this research project contact us as soon as possible. We will help to arrange appropriate medical treatment for your child. If your child becomes ill and requires hospital treatment, if possible they should be taken to Children's Hospital Number 1 or 2. All children participating in the study will be covered by vaccine trial insurance.

Will we be informed of the results when the research project is finished?

We will send you a letter about the overall results at the end of the study.

How is the study funded?

This study is funded by the Bill & Melinda Gates Foundation. The sponsor is the Murdoch Childrens Research Institute, Melbourne, Australia.

Ethical Approval

This study has been approved by the People's Committee of Ho Chi Minh City. This study has also been approved by the Vietnam Ministry of Health Ethics Committee and by The Royal Children's Hospital Melbourne Human Research Ethics Committee. The ethics committees make sure that the study is being done in the best and safest way. If you have any concerns or complaints regarding the conduct of the research project you are invited to contact:

Vietnam Ministry of Health Ethics Committee<br>Phone: 04 62732156	OR	Director, Research Ethics & Governance,<br>The Royal Children's Hospital Melbourne<br>Phone: +61 3 9345 5044

Who should I contact for more information?

Please feel free to contact us if you would like more information about the project or if you need to speak to a member of the research team in an emergency.

If you have any questions regarding the study activities, please phone Dr Tran Phuc Hau: 0904473899

If you have any questions regarding adverse events, please phone study doctors at the site. Contact details are included in the Parent Held Record.

For peer review only - http://bmjopen.bmj.com/site/about/guidelines.xhtml

PART II: CONSENT FORM

Participant ID: |__||__||__||__|

Research Project Title: **Vietnam Pneumococcal Trial II**

HREC Project Number: 36027

- I have read, or someone has read to me in a language that I understand, the information statement version listed above and I understand its contents.
- I believe I understand the purpose, extent and possible risks of my child's involvement in this project.
- I voluntarily consent for my child to take part in this research project.
- I have had an opportunity to ask questions and I am satisfied with the answers I have received.
- I understand that this project has been approved by Vietnam Ministry of Health Ethics Committee and The Royal Children's Hospital Melbourne Human Research Ethics Committee and will be carried out in line with the National Statement on Ethical Conduct in Human Research (2007) – including all updates.
- I understand I will receive a copy of this Information Statement and Consent Form.

CONSENT

☐ I do	☐ I do not	agree for my baby to take part in this study

USE OF SAMPLES

☐ I do	☐ I do not	consent to the storage of my child's unused blood/NP samples for future work in the same general area of research that has obtained ethics committee approval

Child's Name Gender: Male / Female Date of birth: ______________

Parent/Guardian Name Parent/Guardian Signature Date
Time: _ _ : _ _

Relationship to Child

If illiterate: *A literate witness must sign (if possible, this person should be selected by the participant and should have no connection to the research team).*

I have witnessed the accurate reading of the consent form to the parent of the potential participant, and the individual has had the opportunity to ask questions. I confirm that the individual has given consent freely.

Witness Name Witness Signature Date

Declaration by researcher: I have explained the project to the parent/guardian who has signed above, and believe that they understand the purpose, extent and possible risks of their child's involvement in this project.

Research Team Member Name Research Team Member Signature Date

Note: All parties signing the Consent Form must date their own signature.

For peer review only - http://bmjopen.bmj.com/site/about/guidelines.xhtml

SPIRIT

STANDARD PROTOCOL ITEMS: RECOMMENDATIONS FOR INTERVENTIONAL TRIALS

SPIRIT 2013 Checklist: Recommended items to address in a clinical trial protocol and related documents*

Section/item	Item No	Description	Addressed on page number
**Administrative information**			
Title	1	Descriptive title identifying the study design, population, interventions, and, if applicable, trial acronym	______1_______
Trial registration	2a	Trial identifier and registry name. If not yet registered, name of intended registry	______2_______
	2b	All items from the World Health Organization Trial Registration Data Set	__Appendix 1___
Protocol version	3	Date and version identifier	__Appendix 1___
Funding	4	Sources and types of financial, material, and other support	______16______
Roles and responsibilities	5a	Names, affiliations, and roles of protocol contributors	______16______
	5b	Name and contact information for the trial sponsor	__Appendix 1___
	5c	Role of study sponsor and funders, if any, in study design; collection, management, analysis, and interpretation of data; writing of the report; and the decision to submit the report for publication, including whether they will have ultimate authority over any of these activities	__Appendix 1___
	5d	Composition, roles, and responsibilities of the coordinating centre, steering committee, endpoint adjudication committee, data management team, and other individuals or groups overseeing the trial, if applicable (see Item 21a for data monitoring committee)	______n/a______

For peer review only

Introduction

Background and rationale	6a	Description of research question and justification for undertaking the trial, including summary of relevant studies (published and unpublished) examining benefits and harms for each intervention	______3-4_____
	6b	Explanation for choice of comparators	______4_______
Objectives	7	Specific objectives or hypotheses	______4-5_____
Trial design	8	Description of trial design including type of trial (eg, parallel group, crossover, factorial, single group), allocation ratio, and framework (eg, superiority, equivalence, noninferiority, exploratory)	______5_______

Methods: Participants, interventions, and outcomes

Study setting	9	Description of study settings (eg, community clinic, academic hospital) and list of countries where data will be collected. Reference to where list of study sites can be obtained	______5_______
Eligibility criteria	10	Inclusion and exclusion criteria for participants. If applicable, eligibility criteria for study centres and individuals who will perform the interventions (eg, surgeons, psychotherapists)	______6_______
Interventions	11a	Interventions for each group with sufficient detail to allow replication, including how and when they will be administered	______6_______
	11b	Criteria for discontinuing or modifying allocated interventions for a given trial participant (eg, drug dose change in response to harms, participant request, or improving/worsening disease)	______7_______
	11c	Strategies to improve adherence to intervention protocols, and any procedures for monitoring adherence (eg, drug tablet return, laboratory tests)	______7_______
	11d	Relevant concomitant care and interventions that are permitted or prohibited during the trial	______7_______
Outcomes	12	Primary, secondary, and other outcomes, including the specific measurement variable (eg, systolic blood pressure), analysis metric (eg, change from baseline, final value, time to event), method of aggregation (eg, median, proportion), and time point for each outcome. Explanation of the clinical relevance of chosen efficacy and harm outcomes is strongly recommended	______7-8_____
Participant timeline	13	Time schedule of enrolment, interventions (including any run-ins and washouts), assessments, and visits for participants. A schematic diagram is highly recommended (see Figure)	______8-9_____

For peer review only - http://bmjopen.bmj.com/site/about/guidelines.xhtml

Sample size	14	Estimated number of participants needed to achieve study objectives and how it was determined, including clinical and statistical assumptions supporting any sample size calculations	______9______
Recruitment	15	Strategies for achieving adequate participant enrolment to reach target sample size	______10______

Methods: Assignment of interventions (for controlled trials)

Allocation:

Sequence generation	16a	Method of generating the allocation sequence (eg, computer-generated random numbers), and list of any factors for stratification. To reduce predictability of a random sequence, details of any planned restriction (eg, blocking) should be provided in a separate document that is unavailable to those who enrol participants or assign interventions	______10______
Allocation concealment mechanism	16b	Mechanism of implementing the allocation sequence (eg, central telephone; sequentially numbered, opaque, sealed envelopes), describing any steps to conceal the sequence until interventions are assigned	______10______
Implementation	16c	Who will generate the allocation sequence, who will enrol participants, and who will assign participants to interventions	______10______
Blinding (masking)	17a	Who will be blinded after assignment to interventions (eg, trial participants, care providers, outcome assessors, data analysts), and how	______10______
	17b	If blinded, circumstances under which unblinding is permissible, and procedure for revealing a participant's allocated intervention during the trial	______n/a______

Methods: Data collection, management, and analysis

Data collection methods	18a	Plans for assessment and collection of outcome, baseline, and other trial data, including any related processes to promote data quality (eg, duplicate measurements, training of assessors) and a description of study instruments (eg, questionnaires, laboratory tests) along with their reliability and validity, if known. Reference to where data collection forms can be found, if not in the protocol	______11______
	18b	Plans to promote participant retention and complete follow-up, including list of any outcome data to be collected for participants who discontinue or deviate from intervention protocols	______11______

For peer review only - http://bmjopen.bmj.com/site/about/guidelines.xhtml

Data management	19	Plans for data entry, coding, security, and storage, including any related processes to promote data quality (eg, double data entry; range checks for data values). Reference to where details of data management procedures can be found, if not in the protocol	______11______
Statistical methods	20a	Statistical methods for analysing primary and secondary outcomes. Reference to where other details of the statistical analysis plan can be found, if not in the protocol	______11-12____
	20b	Methods for any additional analyses (eg, subgroup and adjusted analyses)	______12______
	20c	Definition of analysis population relating to protocol non-adherence (eg, as randomised analysis), and any statistical methods to handle missing data (eg, multiple imputation)	______12______
**Methods: Monitoring**			
Data monitoring	21a	Composition of data monitoring committee (DMC); summary of its role and reporting structure; statement of whether it is independent from the sponsor and competing interests; and reference to where further details about its charter can be found, if not in the protocol. Alternatively, an explanation of why a DMC is not needed	______12______
	21b	Description of any interim analyses and stopping guidelines, including who will have access to these interim results and make the final decision to terminate the trial	______12______
Harms	22	Plans for collecting, assessing, reporting, and managing solicited and spontaneously reported adverse events and other unintended effects of trial interventions or trial conduct	______13______
Auditing	23	Frequency and procedures for auditing trial conduct, if any, and whether the process will be independent from investigators and the sponsor	______13______
**Ethics and dissemination**			
Research ethics approval	24	Plans for seeking research ethics committee/institutional review board (REC/IRB) approval	______13______
Protocol amendments	25	Plans for communicating important protocol modifications (eg, changes to eligibility criteria, outcomes, analyses) to relevant parties (eg, investigators, REC/IRBs, trial participants, trial registries, journals, regulators)	______14______

Consent or assent	26a	Who will obtain informed consent or assent from potential trial participants or authorised surrogates, and how (see Item 32)	______14______
	26b	Additional consent provisions for collection and use of participant data and biological specimens in ancillary studies, if applicable	______14______
Confidentiality	27	How personal information about potential and enrolled participants will be collected, shared, and maintained in order to protect confidentiality before, during, and after the trial	______14______
Declaration of interests	28	Financial and other competing interests for principal investigators for the overall trial and each study site	______15______
Access to data	29	Statement of who will have access to the final trial dataset, and disclosure of contractual agreements that limit such access for investigators	______15______
Ancillary and post-trial care	30	Provisions, if any, for ancillary and post-trial care, and for compensation to those who suffer harm from trial participation	______15______
Dissemination policy	31a	Plans for investigators and sponsor to communicate trial results to participants, healthcare professionals, the public, and other relevant groups (eg, via publication, reporting in results databases, or other data sharing arrangements), including any publication restrictions	______15______
	31b	Authorship eligibility guidelines and any intended use of professional writers	______15______
	31c	Plans, if any, for granting public access to the full protocol, participant-level dataset, and statistical code	______15______
**Appendices**			
Informed consent materials	32	Model consent form and other related documentation given to participants and authorised surrogates	__Appendix 3___
Biological specimens	33	Plans for collection, laboratory evaluation, and storage of biological specimens for genetic or molecular analysis in the current trial and for future use in ancillary studies, if applicable	__Appendix 2___

*It is strongly recommended that this checklist be read in conjunction with the SPIRIT 2013 Explanation & Elaboration for important clarification on the items. Amendments to the protocol should be tracked and dated. The SPIRIT checklist is copyrighted by the SPIRIT Group under the Creative Commons "Attribution-NonCommercial-NoDerivs 3.0 Unported" license.
